# Efficient Integration of 5‐Hydroxymethylfurfural Oxidation to 2,5‐Furandicarboxylic Acid with Electrochemical Reduction of CO_2_ to Tunable Syngas Production in a Flow Cell

**DOI:** 10.1002/cssc.202502122

**Published:** 2025-10-16

**Authors:** Moritz Lukas Krebs, Anil Kumar Sihag, Eko Budiyanto, Harun Tüysüz, Christian M. Pichler, Ferdi Schüth

**Affiliations:** ^1^ Department of Heterogeneous Catalysis Max‐Planck‐Institut für Kohlenforschung 45470 Mülheim an der Ruhr Germany; ^2^ Center for Electrochemical Surface Technology GmbH 2700 Wiener Neustadt Austria; ^3^ Institute of Applied Physics Vienna University of Technology 1040 Vienna Austria; ^4^ Catalysis and Energy Materials IMDEA Materials Institute 28906 Getafe Madrid Spain

**Keywords:** 5‐hydroxymethylfurfural oxidation reaction, CO_2_ electroreduction, flow cell, gas diffusion electrodes, syngas

## Abstract

Pairing electrochemical CO_2_ reduction (CO_2_RR) with the oxygen evolution reaction (OER) significantly limits overall system efficiency due to the high energy demand of the OER and low product value. Here, a scalable electrochemical platform is present that couples CO_2_RR with the oxidation of 5‐hydroxymethylfurfural (HMF) to the high‐value product 2,5‐furandicarboxylic acid (FDCA). Using a bimetallic FeCo‐modified Ni‐anode, prepared via a Fenton‐like surface treatment, achieves >95% FDCA yield and Faradaic efficiency under industrially relevant conditions by oxidizing stable Cannizzaro‐derived intermediates. Integration with CO_2_RR in an electrochemical flow cell enables syngas production with tunable H_2_/CO ratios (0.1–4) and >92% overall Faradaic efficiency. Simultaneously, FDCA is produced at the anode with ≈89% Faradaic efficiency and yields exceeding 90%. Economic analysis indicates an 11–12% improvement in overall energy efficiency, with FDCA contributing more than 96% of the system revenue. This work establishes a scalable, energy‐efficient platform for concurrent CO_2_ utilization and biomass upgrading, advancing sustainable electrochemical production.

## Graphical abstract

An integrated electrochemical system combines CO_2_ reduction (CO_2_RR) with 5‐hydroxymethylfurfural (HMF) oxidation reaction (HMFOR) to produce syngas (H_2_/CO) and 2,5‐furandicarboxylic acid (FDCA). A FeCo‐modified Ni‐anode enables ≈90% FDCA yield and Faradaic efficiency in 5 M KOH. Simultaneously, CO_2_RR delivers tunable syngas ratios and >92% Faradaic efficiency, improving both energy and economic performance of the system.

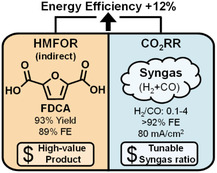

## 1 Introduction

The steady rise in atmospheric CO_2_ concentrations due to the extensive use of fossil fuels has become a critical environmental challenge, driving the urgent need for sustainable carbon mitigation strategies.^[^ ^1^, ^2^ ^]^ Among various approaches, the electrochemical reduction of CO_2_ (CO_2_RR) has emerged as a promising solution for carbon capture and utilization, enabling the conversion of CO_2_ into valuable chemicals and fuels.^[^ ^3^, ^4^, ^5^ ^]^ Over the last decade, the production of CO, HCOOH, and multicarbon (C_2+_) products from CO_2_RR in aqueous medium has attracted much research interest.^[^ ^6^, ^7^ ^]^ In aqueous environments, the hydrogen evolution reaction (HER) occurs as a competing reaction in the same potential range.^[^ ^8^, ^9^, ^10^ ^]^ This can be exploited for the production of syngas (a mixture of H_2_ and CO) as a central intermediate for the chemical industry.^[^ ^11^, ^12^, ^13^, ^14^ ^]^ For example, syngas is extensively used in Fischer–Tropsch synthesis to produce long‐chain hydrocarbons, requiring an optimal H_2_/CO ratio of ≤2:1. Also, syngas is critical for methanol production, where an H_2_/CO ratio of ≥2:1 is desirable, while dimethyl ether synthesis typically requires a 1:1 ratio.^[^ ^15^, ^16^, ^17^ ^]^ However, syngas production through coal gasification, natural gas reforming, or the reverse water–gas shift reaction of captured CO_2_ is energy‐intensive, requires harsh conditions, and generates significant CO_2_ emissions, contradicting the principles of green chemistry.^[^ ^3^, ^16^, ^17^ ^]^ Electrocatalytic conversion of CO_2_ to syngas offers a sustainable alternative that operates under milder conditions while enabling tunable H_2_/CO ratios tailored to specific industrial applications.^[^ ^3^, ^4^, ^14^ ^]^

Despite advancements in catalyst development for CO_2_RR to achieve tunable syngas compositions, challenges persist in scaling up these systems while maintaining precise control over H_2_/CO ratios at high current densities.^[^ ^14^ ^]^ Additionally, the oxygen evolution reaction (OER), commonly paired with the CO_2_RR as the counter reaction in an electrolyzer, suffers from sluggish kinetics and high overpotentials while the produced oxygen is of low economic value. OER can consume up to 94.5% of the input energy in such systems, significantly limiting their overall energy efficiency.^[^ ^18^, ^19^ ^]^ To address this limitation, replacing OER with anodic reactions that yield valuable oxidative products has gained attention as a strategy to enhance system efficiency.^[^ ^19^, ^20^, ^21^ ^]^ For instance, Verma et al.^[^ ^22^ ^]^ demonstrated that coupling CO_2_RR with glycerol oxidation can reduce energy input by up to 53%, while Liu et al.^[^ ^23^ ^]^ reported that replacing OER with the 5‐hydroxymethylfurfural (HMF) oxidation reaction (HMFOR) improved the energy efficiency of formate production by ≈23%. The electrochemical oxidation of biomass‐derived HMF to 2,5‐furandicarboxylic acid (FDCA) is of particular interest as FDCA is a key precursor for the production of biopolymers such as polyethylene furanoate (PEF), an emerging sustainable substitute for polyethylene terephthalate (PET).^[^ ^24^ ^]^ To date, however, only a few studies have investigated the integration of the HMFOR with the CO_2_RR in a flow electrolyzer. Moreover, in integrated HMFOR–CO_2_RR systems, most studies report either high Faradaic efficiencies (FE) or (carbon) yields (>90%) for FDCA production, but seldom demonstrate excellence in both, independent, key performance metrics.^[^ ^23^, ^25^, ^26^, ^27^, ^28^, ^29^, ^30^ ^]^ For implementing a truly meaningful process, it is essential that both parameters, FE and carbon yield, are optimized. A significant challenge in the electrooxidation of HMF lies in its intrinsic instability under highly alkaline conditions (pH ≥ 13), which, while promoting its conversion to FDCA, also accelerates its degradation into polymeric species (humins), leading to substantial carbon losses.^[^ ^31^, ^32^ ^]^ For electrochemical flow cells, this instability gives rise to a fundamental trade‐off between achieving high FDCA yields and high Faradaic efficiency, as elevated current (density)—required to minimize the residence time of unstable HMF in the alkaline electrolyte—also increases competition with the parasitic OERs at more elevated anodic potentials.^[^ ^25^, ^33^ ^]^

To address current challenges for coupling HMFOR with CO_2_RR in flow systems, specifically, achieving high FDCA yields while maintaining high FE for both the CO_2_RR and HMFOR, we present a three‐step strategy that enables efficient HMFOR under strongly alkaline conditions, while simultaneously allowing integration with CO_2_RR for tunable syngas production. First, we develop and apply a novel and improved bimetallic FeCo‐modified Ni‐anode by extending a previously reported synthesis method to enable bimetallic surface modification.^[^ ^34^ ^]^ Specifically, we apply a Fenton‐like treatment using H_2_O_2_ and FeCl_3_ to chemically modify a nickel foam (NF) in the presence of CoCl_2_. Structural and electrochemical characterization revealed improved performance and selectivity of the bimetallic modified Ni‐anode toward the HMFOR. Second, we demonstrate that the improved Ni‐anode is ideally suited for the electrooxidation of Cannizzaro‐derived HMF intermediates, namely 5‐hydroxymethyl‐2‐furancarboxylic acid (HMFCA) and 5,5‐dihydroxymethylfuran (DHMF), which can be used as stable HMF equivalents in the alkaline electrooxidation to FDCA. In consequence, we display that this strategy allows the indirect oxidation of HMF at elevated concentrations of both HMF (500 mM) and KOH (5 M). Finally, we scale up and integrate this system with the CO_2_RR in a semi‐batch flow mode, enabling the production of tunable H_2_/CO syngas mixtures. We report H_2_:CO ratios ranging from 0.1 to 4 while maintaining high FE (>92%) for gaseous products, while producing FDCA with FE of 89% and yields of 93%. This is one of the very few HMFOR systems that simultaneously achieve high FE and carbon balance (CB). To our knowledge, this is also the first report of achieving controllable and tunable syngas ratios while coupling the CO_2_RR with the HMFOR.

## 2 Results and Discussion

### 2.1 Preparation and characterization of the anode

**Figure 1:**
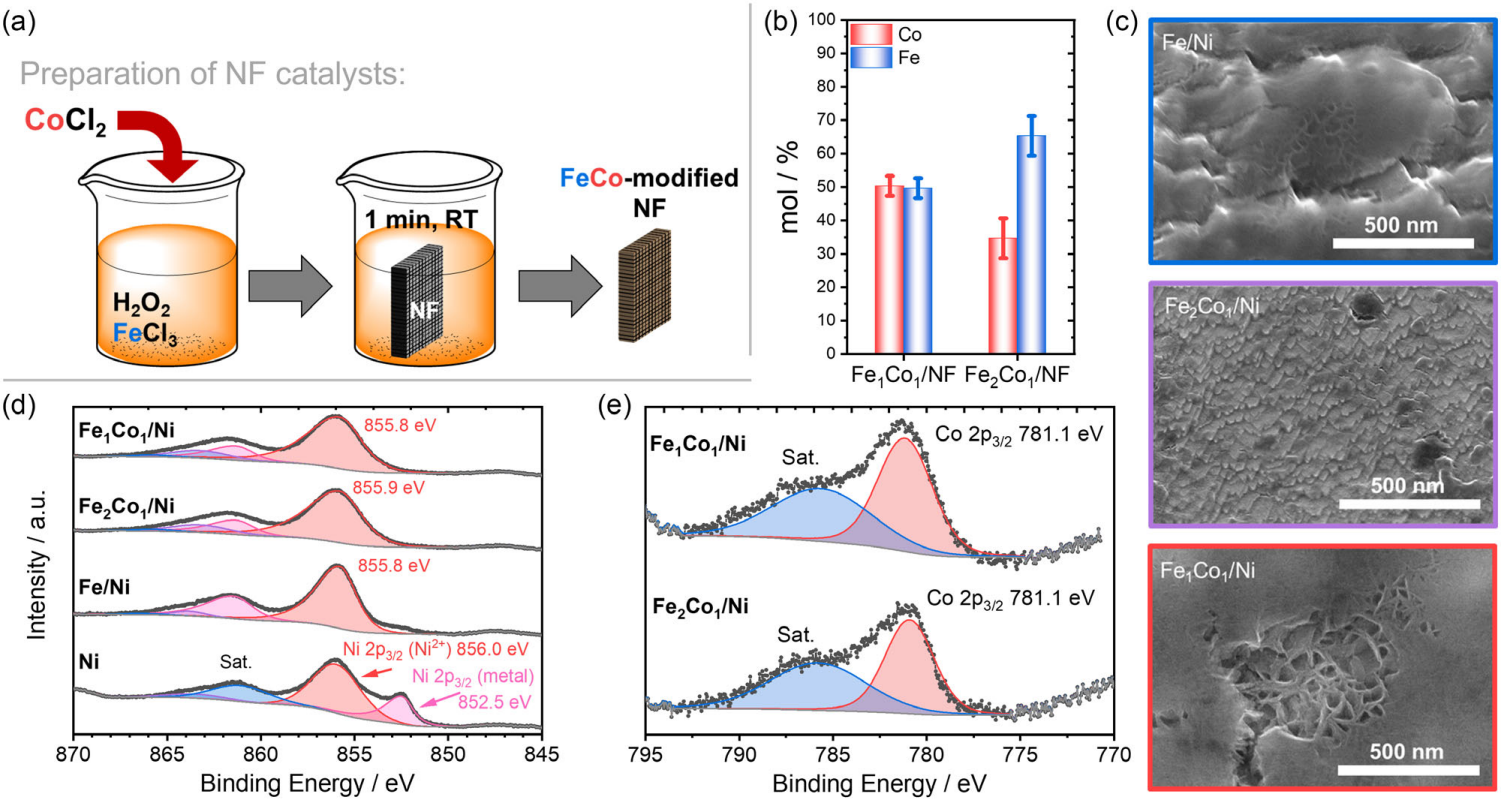
a) Schematic illustration of the modification process to yield transition metal modified NF electrodes. b) ICP‐OES results for the as‐prepared FeCo‐modified NF compared to the nominal metal ratio. c) SEM images after surface modification. d) Ni 2*p* XPS spectra of the Ni substrates treated in H_2_O_2_ in the presence and absence of the respective transition metals. e) Co 2*p* XPS spectra confirming surface loading of the FeCo‐modified NF materials.

The FeCo‐modified Ni foam (Fe_1_Co_1_/NF) catalysts and the Fe‐modified Ni foam reference catalyst (Fe/NF) were synthesized via a modified Fenton‐like reaction involving Fe^3+^ precursors and H_2_O_2_, as previously developed in our laboratory (i.e., the Max‐Planck‐Institute für Kohlenforschung) by Changlong Wang.^[^ ^34^ ^]^ 30 mL of a 5 wt% H_2_O_2_ solution was mixed with the desired amounts of transition metals and allowed to react for 5 min. A Ni foam or Ni sheet (3 × 1 cm) was then immersed in this transition metal–peroxide mixture and removed precisely after 1 min. The Ni substrate was subsequently rinsed thoroughly with deionized water and dried at 60 °C in ambient air for 24 h, as schematically illustrated in **Figure** **1**a (see the Supporting Information for more details). The elemental composition of the synthesized FeCo/NF was determined by inductively coupled plasma optical emission spectrometry (ICP‐OES) (Figure 1b), confirming the deposition of Fe and Co at the desired loading ratio. Scanning electron microscopy (SEM) imaging of the catalyst surface (Figure 1c) revealed increased surface roughness across all modified Ni catalysts, though no significant morphological alterations were observed. This finding is consistent with grazing incidence X‐ray diffraction (GIXRD) analysis, which showed no detectable reflexes corresponding to crystalline Fe, Ni, or Co phases on the Ni surface (Figure S2, Supporting Information). The absence of such reflexes in the GIXRD patterns suggests the formation of an amorphous or poorly crystalline surface layer, which cannot be detected by GIXRD methods. To further probe the surface composition, X‐ray photoelectron spectroscopy (XPS) analysis was conducted (Figure S3, Supporting Information). A comparison of the Ni 2*p* spectra (Figure 1d) demonstrated that the H_2_O_2_‐treated Ni substrate retained some metallic character, indicated by the presence of a peak at 852 eV. Treatment with a mixture of H_2_O_2_ and the respective transition metals resulted in increased surface oxidation, as evidenced by the diminished signal for metallic Ni. Analysis of the Co 2*p* spectra (Figure 1d) also confirmed the presence of Co^2+^ species on the catalyst surface. From the Fe 2*p* spectra (Figure S4, Supporting Information), we identified that Fe is deposited on the surface of all Fe‐containing samples, likely in both Fe^2+^ and Fe^3+^ oxidation states.

### 2.2 Electrochemical testing of the anode

**Figure 2:**
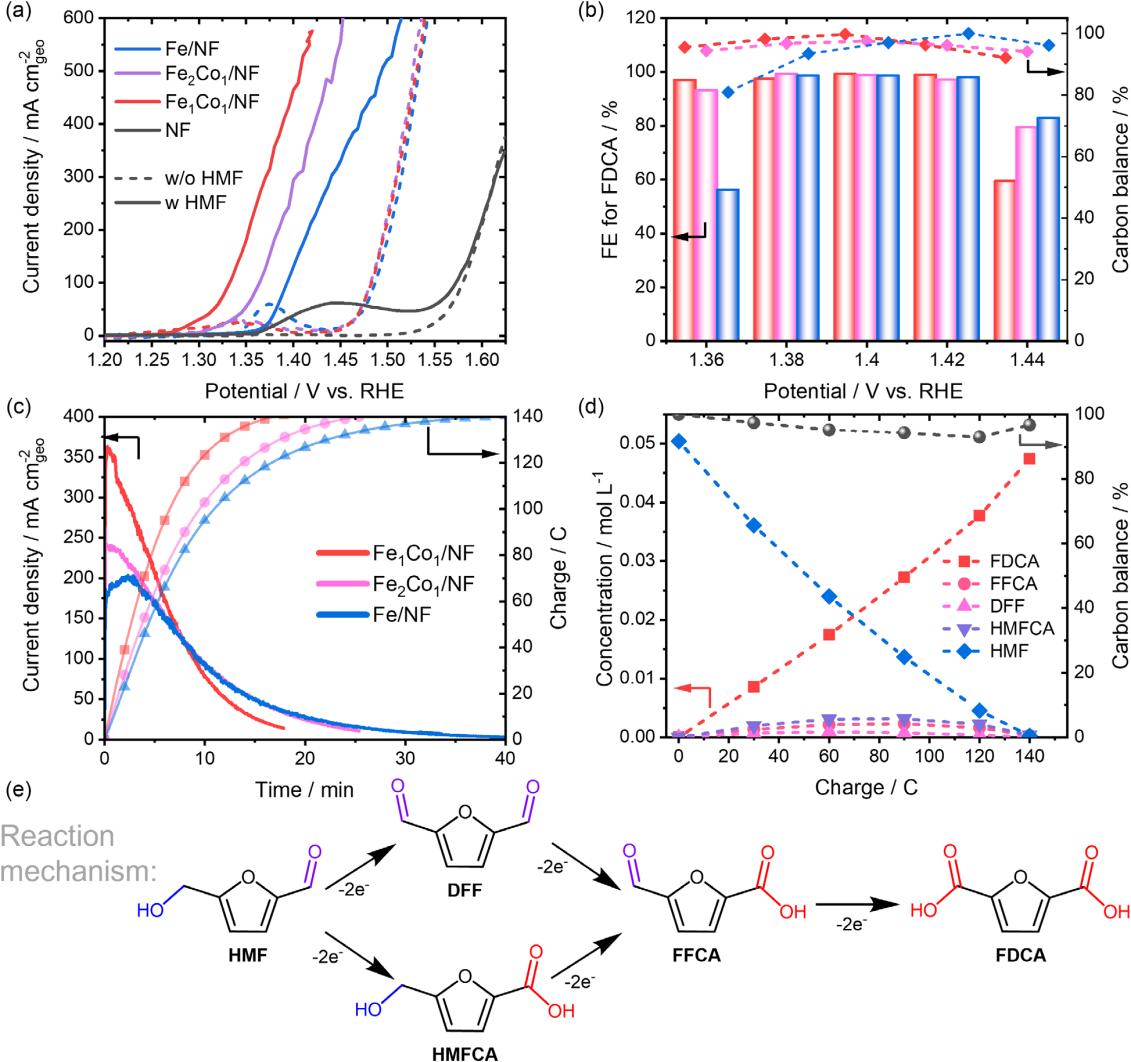
a) LSV results for the H_2_O_2_‐treated NF electrodes in the presence (solid line) and absence (dotted line) of 50 mM HMF. Experiments were conducted in 1 M KOH (Scan rate: 5 mV s^−1^; potential is not iR corrected). b) Faradaic efficiency (FE) of FDCA production for different modified NF catalysts in CA measurements at various potentials, applying 140 C of charge to the solution. c) Exemplary CA measurements at 1.40 V vs. RHE. d) Product formation as a function of injected charge using the Fe_1_Co_1_/NF catalyst in 1 M KOH with 50 mM HMF. e) Reaction mechanism of HMF oxidation.

The electrochemical performance of the modified Ni foam catalysts was assessed via linear sweep voltammetry (LSV) in 1 M KOH in a batch cell setup, both with and without 50 mM HMF (**Figure** **2**a). Among the tested samples, the Fe_1_Co_1_/NF catalyst exhibited the highest activity, achieving a current density of 300 mA cm^−2^ at 1.37 V vs. reversible hydrogen electrode (RHE). This performance ranks among the most active catalysts for the HMFOR reported in the literature (Figure S5, Supporting Information). The catalytic activity correlates with the Fe/Co ratio, as Fe_2_Co_1_/NF and Fe/NF required higher potentials of 1.40 and 1.44 V vs. RHE to reach 300 mA cm^−2^, respectively (Figure S5, Supporting Information). Notably, the addition of Co had no significant effect on the OER activity, extending the potential window for HMFOR from 80 mV (Fe/NF) to ≈150 mV (Fe_1_Co_1_/NF) in the presence of 50 mM HMF. Yet, the enhanced performance of the Co‐containing samples was accompanied by a shift in the pre‐OER signal, associated with the oxidation of Ni^2+^ to Ni^3+^ at lower potentials. This finding is in line with the proposed reaction mechanism for Ni catalysts in alcohol and aldehyde electrooxidation in highly alkaline electrolytes, proceeding via the formation of redox‐active Ni^3+^ (indirect mechanism) or Ni^4+^ species (potential‐dependent mechanism).^[^ ^35^, ^36^, ^37^ ^]^

Chronoamperometry (CA) measurements (Figure 2b,c) were conducted to analyze the efficiency of the different working electrodes in the HMFOR to FDCA (Figure S6, Supporting Information). The Fe_1_Co_1_/NF catalyst demonstrated FE approaching 100% across a potential range of 1.36–1.42 V vs. RHE, with an almost complete CB of >95%. This proves the high activity and selectivity of the CoFe‐modified electrodes in HMFOR over an extended potential regime. In contrast, the Fe/NF electrode exhibited comparable performance only between 1.40 and 1.42 V vs. RHE, with significantly reduced activity at lower potentials. At 1.38 V vs. RHE, both the CB and FE significantly decline. This decrease in performance is likely due to the non‐Faradaic conversion of HMF and its intermediates by condensation reactions facilitated by the aldehyde functionality, resulting in polymeric species, commonly referred to as humins.^[^ ^31^, ^32^ ^]^ Yet even at 1.40 V vs. RHE, the Fe/NF catalyst requires ≈35 min to achieve full HMF conversion, whereas the Fe_1_Co_1_/NF catalyst requires just 20 min (Figure 2d). As the primary reaction intermediates, HMFCA and 5‐formyl‐2‐furancarboxylic acid (FFCA), were detected when using the Fe_1_Co_1_/NF catalyst (Figure 2d). This suggests that the HMFOR proceeds predominantly via the HMFCA pathway rather than through 2,5‐diformylfuran (DFF) formation (Figure 2e and S7, Supporting Information). This observation aligns with previous literature indicating preferential oxidation of the aldehyde functionalities under strongly alkaline conditions, likely facilitated by equilibrium with the geminal diol.^[^ ^38^, ^39^ ^]^

Aside from high activity and efficiency in the oxidation of HMF, the stability of the electrodes is crucial. Catalyst stability was assessed by performing five successive HMF oxidation cycles at 1.40 V vs. RHE. Across all cycles, FE and CB values remained above 95% for Fe_1_Co_1_/NF and Fe_2_Co_1_/NF (Figure S8, Supporting Information), indicating sustained activity and selectivity. Further chronopotentiometry (CP) tests conducted over 24 h at 500 mA cm^−2^ in 1 M KOH (Figure S9, Supporting Information) revealed no significant loss in OER activity over the course of the experiment. Post‐experimental LSV and CA (Figure S9, Supporting Information) measurements in the presence of HMF confirmed that the catalysts also retained their high activity as well as selectivity in the HMFOR. Post‐characterization of the spent material by ICP‐OES revealed that the Fe/Co ratio remained stable for the FeCo‐modified catalysts even when treated under harsh reaction conditions (Figure S10, Supporting Information). Yet, SEM imaging indicated the formation of sheet‐like structures on the catalyst surface, suggesting a surface reconstruction process likely driven by the formation of Ni(OH)_2_/NiOOH species under applied bias, in line with the literature.^[^ ^40^, ^41^ ^]^ This hypothesis is further validated by XPS analysis, confirming the presence of Ni^2+^ species on the surface of the spent catalysts (Figure S10, Supporting Information).

**Figure 3:**
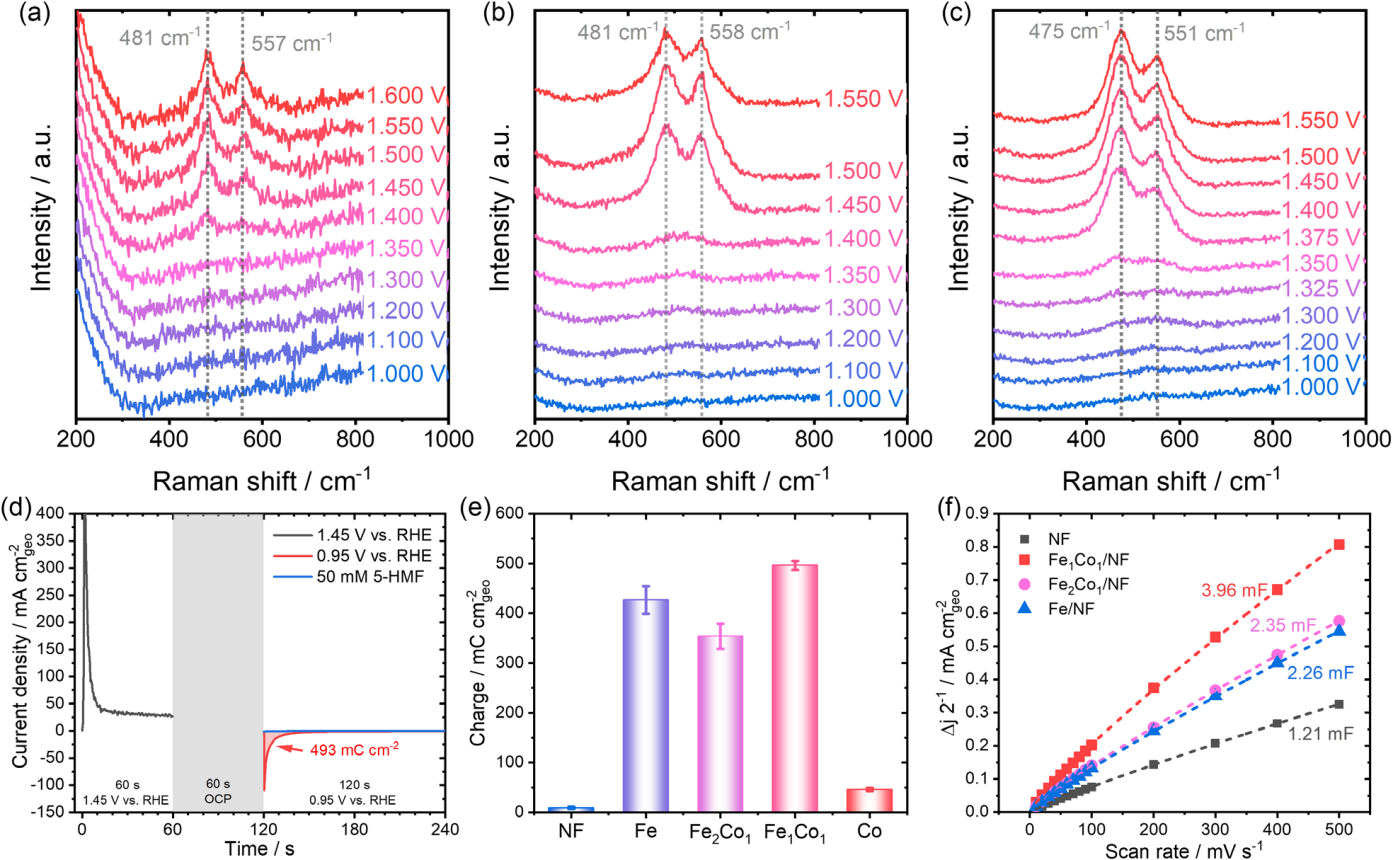
a–c) In situ Raman analysis of H_2_O_2_‐treated Ni substrates under increasing potentials in 0.1 M KOH. (a) H_2_O_2_‐treated Ni sheet. (b) Fe/Ni sheet. (c) Fe_1_Co_1_/Ni sheet. d) Exemplary pulse voltammetry measurements for the Fe_1_Co_1_/NF sample to assess the redox properties of the electrode. Reductive charge was measured in the presence (blue) and absence (red) of 50 mM HMF in 1 M KOH. Before applying a reductive potential (0.95 V vs. RHE), the sample was oxidized at 1.45 V vs. RHE for 1 min. e) Reductive charge measured via pulse voltammetry at 0.95 V vs. RHE in the absence of HMF for different electrodes, demonstrating the impact of Fe and H_2_O_2_ treatment on the redox activity of the catalyst surface. f) Double‐layer capacitance (C_dl_) measurements for different NF materials.

To gain deeper insight into the influence of Co on the redox chemistry of the Ni surface, in situ Raman spectroscopy was conducted at oxidative potentials in 0.1 M KOH. Raman measurements of the H_2_O_2_‐treated Ni substrate showed no initial Raman bands (**Figure** **3**a). Starting at ≈1.40 V vs. RHE, and becoming more pronounced at higher potentials, a weak pair of Raman signals appeared at 481 and 557 cm^−1^. These bands correspond to the E_g_ mode (Ni–O bending vibrations within the oxygen plane) and the A_1g_ mode (within the oxygen plane) vibration of NiOOH, respectively.^[^ ^40^, ^41^, ^42^ ^]^ The formation of NiOOH indicates surface oxidation of the Ni electrode, which is particularly important, as higher‐valent Ni species are known to be the active species that facilitate the oxidation of alcohols and aldehydes on Ni electrodes.^[^ ^36^, ^37^ ^]^ Treating the Ni substrate with Fe and H_2_O_2_ (Figure 3b) did not result in the appearance of initial Raman bands but led to more intense NiOOH vibrations—indicated by the much increased signal‐to‐noise ratio—accompanied by a visible color change to a deep black color (Figure S11, Supporting Information). By the addition of Co, a change in the peak intensity ratio of the E_g_ and A_1g_ bands (Figure S12, Supporting Information) is observed. The change in the intensity of these bands is associated with modifications in the local Ni–O structure and may be attributed to a phase transition from β‐like to γ‐like NiOOH induced by the addition of Co (Figure 3c).^[^ ^41^, ^42^, ^43^, ^44^ ^]^ Moreover, the Raman peaks corresponding to the NiOOH phase started to evolve at potentials as low as 1.35 V vs. RHE, consistent with the shift of the pre‐OER signal detected during the LSV measurements (Figure 2a). Also, Co addition resulted in a slight shift in the E_g_ band from 481 to 473 cm^−1^ and the A_1g_ band from 557 to 553 cm^−1^ of the formed NiOOH phase (Figure S13, Supporting Information). This shift can be attributed to Ni—O bond elongation, as previously reported for both Co‐ and Fe‐substituted Ni layered double hydroxides.^[^ ^45^, ^46^ ^]^ These observations suggest that the introduction of Co leads to the formation of a Co(Fe)‐doped NiOOH phase, promoting Ni surface oxidation and thereby enhancing HMFOR performance at lower potentials.^[^ ^47^, ^48^, ^49^ ^]^ To confirm that the NiOOH species in fact serve as the active species in HMFOR, additional measurements were conducted in the presence of HMF. The results indicate that HMF suppresses the formation of NiOOH phases through a spontaneous reaction with the oxidized Ni surface (Figure S14, Supporting Information).^[^ ^35^, ^36^ ^]^ This spontaneous reaction is further evidenced by the rapid decrease in the intensity of Raman bands as soon as HMF is introduced to the oxidized surface under open‐circuit potential (OCP) conditions (Figure S15, Supporting Information). Notably, this surface reduction is also accompanied by a gradual color change of the catalyst from deep black to a brownish appearance, visualizing the reduction of the oxidized Ni surface in the presence of HMF.

The findings from Raman spectroscopy are further supported by pulse voltammetry (Figure 3d,e), which was used to compare the formation of redox‐active Ni species across different catalysts (Figure S16, Supporting Information).^[^ ^36^, ^50^, ^51^ ^]^ In the absence of HMF, oxidation of the catalyst surface was confirmed by a reductive current measured upon applying 0.95 V vs. RHE after a 1 min resting period under open‐circuit potential (OCP; here referring to open‐circuit conditions). However, the introduction of HMF during the resting step drastically reduced this charge by several orders of magnitude, reinforcing the notion that HMF reacts spontaneously with the oxidized surface. Consequently, the reductive charge measured in the absence of HMF serves as an indicator for comparing the number of redox‐active species on the catalyst surface that are accessible for HMF oxidation. Herein, it is shown that activation with Fe and H_2_O_2_, independent of the presence of the Co precursor, results in a two‐order‐of‐magnitude increase in reductive charge compared to the H_2_O_2_‐treated Ni foam (Figure 3e). A similar effect by the introduction of Fe has been observed in previous studies on other Ni‐based catalyst systems.^[^ ^51^, ^52^ ^]^ In contrast, treatment of the NF with Co and H_2_O_2_, in the absence of Fe, resulted in a significantly reduced charge, further validating that the combination of Fe and H_2_O_2_ is essential for the elevated current densities measured for the NF samples. This result also indicates that Co doping primarily affects the surface redox properties, as evidenced by Raman spectroscopy, but has a limited effect on the kinetic properties of the NF catalyst when compared to the Fe activation.^[^ ^53^, ^54^ ^]^ Moreover, the presence of Fe might also promote the formation of high‐valent cobalt species in the FeCo‐modified catalysts, reported to exhibit faster oxidation kinetics in the HMFOR than Co^3+^ species formed at lower potentials.^[^ ^53^, ^55^ ^]^ Measurements of the double‐layer capacitance (C_dl_; Figure S17, Supporting Information) indicate that changes in the electrochemically active surface area do not significantly influence the observed trends. As shown in Figure 3f, a comparison between transition metal modified and H_2_O_2_ treated NF electrodes reveals only a slight variation in C_dl_, which therefore cannot be responsible for the observed change in the reductive charge by orders of magnitude.

**Figure 4:**
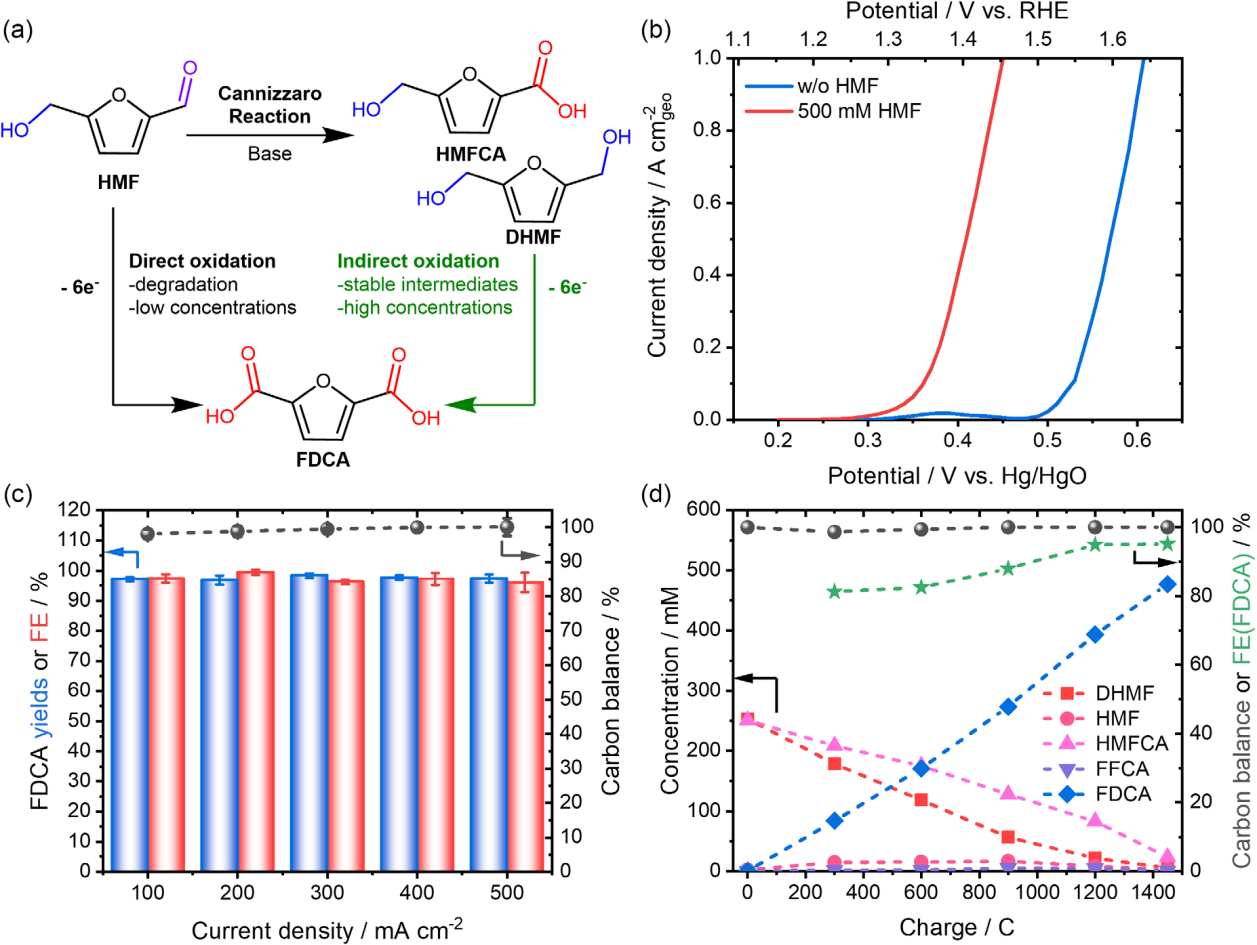
a) Schematic illustrating the advantages of the indirect HMFOR approach via the Cannizzaro reaction. b) LSV results for the Fe_1_Co_1_/NF electrode in 5 M KOH, measured in the presence and absence of 500 mM Cannizzaro products (scan rate: 5 mV s^−1^; potential is not iR‐corrected). Potentials are also reported against the Hg/HgO reference, as conversion to RHE is highly dependent on OH^−^ activity, which may be affected by high salt concentrations and the elevated concentration of organic compounds. c) FE yields and CB obtained from CP measurements in 5 M KOH with 500 mM Cannizzaro products at various current densities. A total charge of 1450 C was passed through into 5 mL of electrolyte using Fe_1_Co_1_/NF electrodes. d) Product formation during the CP measurements at 500 mA cm^−2^ using the Fe_1_Co_1_/NF electrode, as a function of injected charge. DFF was not detected under the applied reaction conditions.

A significant challenge in the transfer of the HMFOR from batch to flow cell operation is presented by the poor stability of HMF in highly alkaline electrolytes, which significantly limits the residence time of the HMF under operating conditions. Yet, electrolysis in less alkaline conditions often results in low selectivity for FDCA and significantly decreased catalytic rates due to the more sluggish regeneration of the oxyhydroxide species and the absence of reactive alkoxide species.^[^ ^53^, ^56^, ^57^ ^]^ We recently demonstrated a solution for this challenge by demonstrating that HMF can be selectively converted via the Cannizzaro reaction into HMFCA and DHMF, both of which are stable even at elevated KOH and substrate concentrations, while also showing increased temperature tolerance.^[^ ^32^ ^]^ Simple conversion of HMF in highly alkaline aqueous media thus enables yields well above 80%, checked in two different laboratories (see Table S1, Supporting Information for reference). Moreover, Gomes et al. reported that even quantitative yields in the Cannizzaro reaction of HMF are achievable through a modified process, demonstrating that the conversion of HMF to stable Cannizzaro products can proceed without carbon loss.^[^ ^58^ ^]^ By design of the Cannizzaro reaction, DHMF and HMFCA are formed in equimolar ratios, allowing for an average 6e^−^ oxidation to FDCA, presenting similar electron efficiency to the electrooxidation of the HMF molecule (**Figure** **4**a). Utilizing this indirect HMFOR concept, we performed electrolysis experiments at 500 mM substrate concentration (250 mM of HMFCA and DHMF each) and 5 M KOH, using the most active Fe_1_Co_1_/NF catalyst. Figure 4b shows that increasing both substrate and KOH concentration results in a stark performance increase, reaching current densities close to 1 A cm^−2^ at a potential decreased by about 150 mV compared to the OER. CP measurements at current densities in the range of 100–500 mA cm^−2^ up to 1450 °C were conducted to further investigate the selectivity of the catalyst under these reaction conditions (Figure 4c). Independent of the applied currents, all experiments demonstrate FE and FDCA yields >95% and a closed CB, indicating that the catalyst is highly selective in the HMFOR of the Cannizzaro products in 5 M KOH. In addition, the experimental results prove that the Cannizzaro products also remain stable, even during prolonged reaction times, as well as at higher current densities and alkalinity of the electrolyte. Product analysis (Figure 4d and S18, Supporting Information) indicates rapid and simultaneous conversion of both Cannizzaro products to FDCA. The hysteresis between HMFCA and DHMF, and the absence of any DFF concentration, further reveal that the oxidation mechanism of DHMF likely proceeds via the formation of HMF and subsequent oxidation to HMFCA, a result in line with previous findings in the HMF electrooxidation in 1 M KOH.

### 2.3 Integration of HMFOR with CO_2_RR in a flow cell

**Figure 5:**
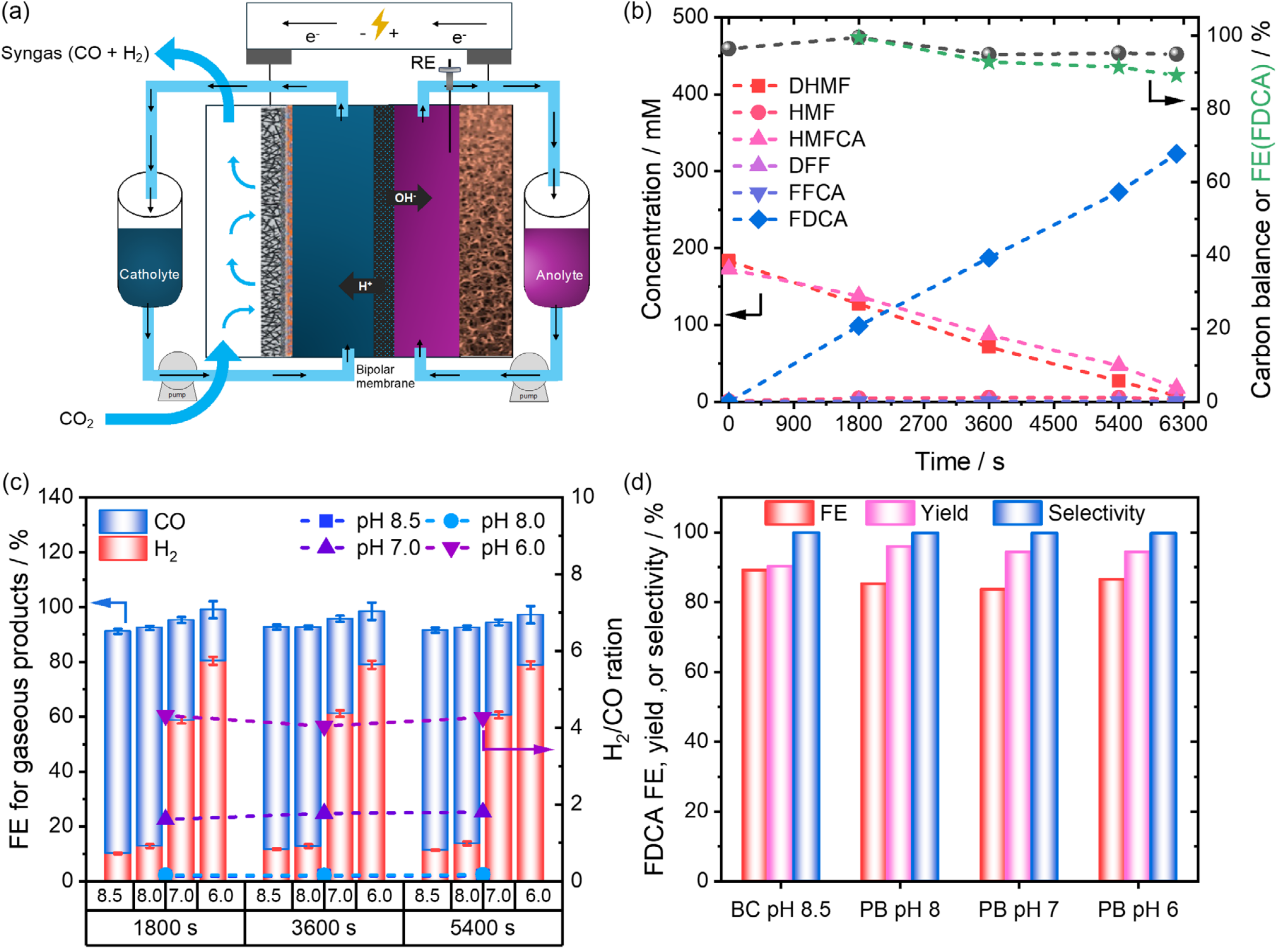
a) Schematic illustration of the continuous‐flow electrolyzer used for the integrated HMFOR–CO_2_RR system. b) Concentrations, FE, and CB of anodic products formed during indirect HMFOR, monitored over time via representative CP measurements at a constant current density of 78.4 mA cm^−2^ using a Fe_1_Co_1_/NF electrode (geometric area: 10.2 cm^2^). Dotted lines are used as guides for the eye. c) FE of cathodic gaseous products (H_2_ and CO) during CO_2_RR on a silver GDE, using either 0.5 M KHCO_3_ (pH 8.5) or a 2 M phosphate buffer at varying pH values. d) Product formation and corresponding FE in indirect HMFOR, performed in 5 M KOH, as a function of the counter electrode configuration used for CO_2_RR. All measurements were conducted at a constant current density of 78.4 mA cm^−2^ under integrated CO_2_RR‐HMFOR coupling conditions.

To demonstrate the scalability of the Cannizzaro approach, the Fe_1_Co_1_/Ni synthesis was scaled to larger electrodes (10.2 cm^2^ area) and tested in a continuous‐flow electrolyzer, which can effectively reduce concentration polarization and enhance mass transfer.^[^ ^59^ ^]^ As illustrated in **Figure** **5**a, the flow electrolyzer composed of three compartments, a gas diffusion chamber, a catholyte chamber, and an anolyte chamber, was utilized for the CO_2_RR‐HMFOR experiments. To prevent product crossover, the anolyte and catholyte chambers were separated by a bipolar membrane. Potentials were measured against an Ag/AgCl leakless electrode. For more details on the experimental setup, refer to the Supporting Information. The LSV curves of Fe_1_Co_1_/Ni revealed that HER‐HMFOR requires an ≈170 mV lower voltage than HER‐OER at 100 mA cm^−2^ current (Figure S19, Supporting Information), comparable to the results in the batch cell. When replacing the HER with the CO_2_RR in a CO_2_RR‐HMFOR system, an even stronger decrease in the cell voltage of up to 900 mV for 80 mA cm^−2^ was observed (Figure S20, Supporting Information), highlighting that HMFOR is clearly preferred on the used catalyst materials. To evaluate the catalyst performance, CP was performed at a constant current of 78.4 mA cm^−2^ (Figure S21–S22, Supporting Information). The electrolysis reaction continued until the complete conversion of HMF was achieved, which occurred after 6200 s, with a total charge of 4960 C passed. Figure 5b illustrates the concentration profiles of the starting materials (HMFCA, DHMF) and intermediates (HMF, DFF, FFCA), as well as the final product (FDCA) over time. After 6200 s of operation, the integrated CO_2_RR–HMFOR system achieved an FDCA yield of 93%, resulting in a selectivity of 99%, and a FE of 89% for FDCA production, corresponding to an average production rate of ≈0.6 g h^−1^. Combined with the closed CB, these results clearly demonstrate that the Cannizzaro approach is transferable from batch to flow electrochemistry.

On the cathode side, a commercial silver catalyst was used that was loaded onto hydrophobic carbon paper with a microporous layer, forming a GDE‐catalyst assembly (see the Supporting Information for more details). As shown in Figure S23, Supporting Information, the system consistently achieved a FE for CO formation of ≈80% when performing the CO_2_RR in a 0.5 M bicarbonate solution at CP at various current densities of 10–100 mA cm^−2^. Only negligible amounts of CH_4_ were formed, consistent with previous reports.^[^ ^14^, ^60^ ^]^ Notably, a maximum CO FE of 83% was obtained at 30 mA cm^−2^, and H_2_ FE ranged between 11% and 15%, with the highest value observed at 100 mA cm^−2^ and the lowest at 10 mA cm^−2^. In addition, the FE for formic acid varied from 2% to 7%, reaching a maximum at 78.4 mA cm^−2^. To tune the catalyst selectivity, the local pH at the cathode–electrolyte interface is critical, as the competing HER is highly sensitive to proton availability.^[^ ^61^, ^62^ ^]^ This principle was used to tune the H_2_:CO ratio in the produced syngas. To stabilize the interfacial pH and enable precise control over proton activity within the pH range of pH 6 to pH 8, we utilized the stronger buffering capacity of phosphate buffer systems near neutrality (pKa_2_ = 7.2). We therefore replaced the 0.5 M KHCO_3_ (pH 8.5) with 2 M potassium phosphate buffers (PB) adjusted to pH 8, pH 7, and pH 6. Electrolysis at pH 8 resulted in increased CO selectivity (H_2_:CO ≈ 0.16), as the higher alkalinity suppresses HER activity. Utilizing a lower pH value of 7 led to an increased H_2_:CO ratio of 1.7. At pH 6, the more acidic environment enhances the HER even further, which, in consequence, shifts the H_2_:CO ratio of the syngas to 4 (Figure 5c). Regardless of the pH, a high FE for gaseous products, exceeding 90%, was maintained under all tested synthesis conditions and remained stable over an extended reaction time of 5400 s. Post‐reaction characterization of the spent GDE/catalyst via SEM further confirmed the catalyst's stability, showing no discernible changes in its properties or morphology (Figure S24–S26, Supporting Information). Additionally, XPS analysis revealed no significant change to the silver catalyst (Figure S27, Supporting Information). Equally important, while the H_2_:CO ratio in the syngas was effectively modulated under varying cathodic conditions, the simultaneous HMFOR maintained consistent performance across all tested conditions (Figure 5d). Overall, this demonstrates the successful integration of both electrochemical processes, enabling high yields, selectivity, and FE for both half‐cell reactions toward valuable products. Especially, the possibility to tune the syngas composition facilitates the implementation of such a process in value chains for the chemical industry, as it allows coupling with various downstream reactions to utilize the formed syngas.

### 2.4 Comparison with Other Approaches and Technical Considerations

Scaling an electrochemical process from batch to flow and subsequently to larger flow reactors introduces several challenges, including mass transfer limitations, electrode degradation, and uneven current distribution. Additional hurdles involve gas–liquid management, pressure control, and ohmic losses that intensify with scale. These challenges are commonly addressed by optimizing cell architecture, employing porous flow‐through electrodes, and integrating modular reactor designs to ensure uniform performance. The gas diffusion electrodes utilized in our study are ideally suited to ensure even gas feed and distribution. For further scaling, constant removal of the FDCA and addition of fresh HMF must be ensured to keep the process running continuously for a longer time. Testing of different electrode configurations (membrane electrode assembly (MEA) vs. GDE, etc.) and establishing continuous product extraction approaches are therefore crucial for the further improvement of this process towards industrial implementation. Despite these complexities, scaling enables enhanced productivity, improved process control, and the feasibility of continuous manufacturing, making it a critical step toward industrial implementation.

Initial considerations reveal that integration of the CO_2_RR coupled with the modified HMFOR system offers significant advantages over the conventional CO_2_RR paired with the OER. The integrated ECO_2_RR‐HMFOR system exhibits significantly higher energy efficiency, lowering the cell voltage by ≈900 mV in a two‐electrode setup compared to the CO_2_RR‐OER system (see SI Figure S24, Supporting Information), at current densities of 78.4 mA cm^−2^. The energy savings by this potential shift are comparable to those demonstrated by Lin et al., resulting in an 11–12% higher energy efficiency compared to the standard CO_2_RR‐OER system, primarily due to its lower energy consumption and improved overall energy utilization.^[^ ^30^ ^]^ This improvement is related to the lower overpotential required for HMFOR, which replaces the energetically demanding OER. According to previous studies, the electrical energy required for electrolysis contributes 50–60% to the total product cost (for CO). A reduction of the required energy input would therefore directly influence the price of the syngas generated.^[^ ^63^, ^64^, ^65^ ^]^

Additionally, HMFOR generates FDCA as a highly valuable product, contributing to economic viability by creating additional revenue streams (with an estimated market price of 1800 $ ton^−1^). It can thus be estimated that the revenue of the FDCA product is significantly higher compared to the cathodic syngas product. This contrasts with the comparatively low syngas price, assumed here to be 200 $ ton^−1^, though actual values vary with feedstock, gas composition, and location.^[^ ^66^, ^67^, ^68^ ^]^ As detailed in the Supporting Information, the annual production of 1002 tons of FDCA is accompanied by the generation of 298 tons of syngas (H_2_:CO = 1:1), yielding a total annual revenue of $1.86 million. Notably, FDCA accounts for over 97% of this revenue. The net present value (NPV), calculated using a cell voltage of 6.2 V at a current density of 80 mA cm^−2^ for the coupled system, and is estimated at $1.44 million. This value increases significantly to $11.7 million when the system is modified to a MEA configuration, which reduces internal resistance and lowers the overall cell voltage (2.7). The transition to an MEA setup is a logical step during further scaling and optimization of process conditions and has been demonstrated previously.^[^ ^23^ ^]^

The sensitivity analysis reveals that the electricity price has a significant impact on operating expenditure (OPEX). Doubling the electricity price results in a 30% increase in the levelized cost of product (LCOP). Another critical factor is the cost of HMF feedstock. We evaluated the sensitivity of the process to variations in HMF feedstock cost over a range of 500–3000 $ per ton. At the base case of 800 $ ton^−1^, the annual HMF input cost is 962,085 $ and the process yields a NPV of $11.74 million. Reducing the HMF price to 500 $ ton^−1^ decreases the HMF input cost by 37.5% and lowers the total OPEX by 30%, resulting in a 61% increase in NPV, up to $18.96 million. In contrast, increasing the HMF price to 1200 $ ton^−1^ raises OPEX by 40% and reduces the NPV by over 80%, to $2.13 million. Beyond $1289/ton, the process becomes economically unviable, with the NPV turning negative. At 2000 and 3000 $ ton^−1^, the NPV falls to −$17.1 million and −$41.2 million, respectively (Table S7, Supporting Information). These results underscore the importance of securing a low‐cost and stable supply of HMF feedstock to maintain the profitability of the system.

Process performance factors such as faradaic efficiency and carbon utilization also influence economic outcomes. The impact of anodic CB on process economics was evaluated over a range from 100% to 20%. At full carbon efficiency, the HMF input cost is $962,085 annually, corresponding to a total OPEX of $1.20 million and an NPV of $11.74 million. As the carbon balance decreases, unconverted HMF increases feedstock consumption and operating costs. For example, at 70% CB, OPEX rises by 24% and NPV drops nearly 50% to $5.97 million. The process becomes economically marginal at 50% CB, where NPV falls to $2.13 million. Below 40% CB, the NPV turns negative, reaching −$3.65 million at 20% CB (Table S8, Supporting Information). These results highlight that maintaining a high carbon balance (≥70%) is critical for sustaining profitability in the coupled CO_2_RR–HMFOR system. Among these, the Faradaic efficiency of FDCA production is particularly important, as FDCA contributes to the vast majority of total revenue.

Advancements in catalyst performance could potentially raise FDCA FE to around 95%, reducing energy consumption and improving overall product yield. Cell voltage is another key factor affecting electricity consumption and thus OPEX. For instance, replacing the OER with HMF oxidation (HMFOR) in the flow cell reduces the cell voltage by 900 mV, resulting in a 29% increase in NPV, as detailed in the Supporting Information. Additionally, reducing the membrane cost from 800 to 200 $ m^−2^ lowers the annual replacement cost by ≈75%, slightly decreasing the LCOP, although this has a relatively minor effect on NPV. These findings align with earlier techno‐economic studies, which highlight the importance of optimizing CO_2_ conversion efficiency and minimizing HMF feedstock costs to achieve economic feasibility for integrated CO_2_RR–HMFOR systems compared to conventional routes where oxygen byproducts provide negligible return.^[^ ^30^ ^]^ These findings highlight the potential of alternative anodic reactions and integrated processes that generate two value‐added products simultaneously, offering a promising path for advancing electrochemical technologies in the chemical industry. In many cases, the anodic products can generate significantly higher revenues. However, it is important to note that in FDCA production, HMF currently accounts for up to 39–77% of the total cost,^[^ ^69^, ^70^ ^]^ which might, however, drastically change if HMF production from biomass is implemented a large scale. Developments focusing on the direct conversion of more complex, but even cheaper waste substrates, might further improve the cost competitiveness of electrochemical processes.^[^ ^71^, ^72^, ^73^ ^]^

Besides the economic feasibility, the sustainability of the process must be considered as well. For electrochemical syngas production, it has been shown that it is with a CO_2_ emission equivalent of 1.5 kg CO_2equiv_ kg^−1^ product less emission intensive compared to state‐of‐the‐art thermochemical processes that emit ≈2.5 kg CO_2eqiv_ kg^−1^ product.^[^ ^74^ ^]^

## 3 Conclusion

We have designed an effective electrochemical strategy that integrates HMFOR and CO_2_RR within a single electrochemical cell. This integrated system not only addresses the energy inefficiencies associated with the OER but also enables the simultaneous production of value‐added products in both half‐cells while maintaining high yields, FE, and selectivities on both electrode sides. At the anode, FDCA is produced with 89% FE and 93% yield. At the cathode, the system allows precise control over syngas composition, achieving H_2_/CO ratios ranging from 0.1 to 4 at relevant current densities of 80 mA cm^−2^, with FE for gaseous products exceeding 92%. Economic considerations indicate that integrating CO_2_RR with the modified HMFOR system improves overall energy efficiency by ≈11–12%. Furthermore, the coproduction of high‐value FDCA alongside syngas significantly enhances economic viability, with FDCA contributing over 97% of total product revenue under continuous operation. However, feedstock costs present a notable challenge for practical implementation, particularly when considering the additional carbon loss associated with the transformation of unstable HMF into stable Cannizzaro products. Although yields exceeding 80% have been achieved across different laboratories and scales, further optimization of the Cannizzaro conversion is necessary to make this approach viable for large‐scale applications. Looking ahead, replacing the Ag catalyst with more advanced Cu‐ or Ni‐based catalysts may also enable the direct production of more valuable C_2+_ products, potentially increasing cathodic revenue. However, the development of efficient and stable catalysts for selective C_2+_ formation remains in its early stages and is not yet competitive with established thermal processes.

## Supporting Information

The authors have cited additional references within the Supporting Information.^[75–97]^

## Conflict of Interest

The authors declare no conflict of interest.

## Supporting information

Supplementary Material

## Data Availability

The data that support the findings of this study are available in the supplementary material of this article.
