# Supplementary material for: Efficient Integration of 5‐Hydroxymethylfurfural Oxidation to 2,5‐Furandicarboxylic Acid with Electrochemical Reduction of CO2 to Tunable Syngas Production in a Flow Cell
Source: ChemSusChem. 2025 Oct 16;18(23):e202502122. doi: 10.1002/cssc.202502122 (PMC12665887; doi:10.1002/cssc.202502122)
Supplement: Supplementary file 1 — Supplementary Material [file CSSC-18-e202502122-s001.pdf]

Supporting Information:

Efficient Integration of 5-Hydroxymethylfurfural Oxidation to 2,5-Furandicarboxylic Acid with Electrochemical Reduction of CO_2 to Tunable Syngas Production in a Flow Cell

Moritz Lukas Krebs†[a], Anil Kumar Sihag†[b,c], Eko Budiyanto[a], Harun Tüysüz[a,d],

Christian M. Pichler[b,c] and Ferdi Schüth[a]*

AUTHOR ADDRESS:

[a]Max-Planck-Institut für Kohlenforschung, 45470 Mülheim an der Ruhr, Germany

[b]Center for Electrochemical Surface Technology GmbH, 2700 Wr.Neustadt, Austria

[c]Institute of Applied Physics, Vienna University of Technology,1040 Vienna, Austria

[d]Catalysis and Energy Materials, IMDEA Materials Institute, 28906 Getafe, Madrid, Spain

†Authors contributed equally.

*To whom correspondence should be addressed:

Email: schueth@kofo.mpg.de

Table of contents

1. Instruments and experimental procedures ... 3

1.1. Chemicals and materials ... 3

1.2. Preparation of electrodes ... 3

Synthesis of Fe-modified Ni substrates: ... 3

Synthesis of FeCo-modified Ni substrates: ... 3

Synthesis of Co-modified Ni substrates: ... 3

H_2O_2 treated Ni substrates: ... 4

Gas diffusion electrode preparation: ... 4

1.3. Electrode characterization ... 4

Inductively Coupled Plasma-Optical Emission Spectrometry (ICP-OES): ... 4

X-Ray photoelectron spectroscopy (XPS) ... 4

Grazing incidence X-ray diffraction (GIXRD) ... 4

Scanning Electron Microscopy (SEM) CEST: ... 5

X-ray photoelectron spectroscopy (XPS) CEST: ... 5

1.4. In-situ Raman measurements: ... 5

1.5. Electrochemical measurements in batch: ... 5

Electrochemical measurements: ... 5

Product analysis: ... 6

1.6. Electrochemical measurements in flow: ... 7

Electrochemical measurements: ... 7

Product analysis: ... 7

1.7. HMF conversion via Cannizzaro reaction ... 8

2. Results ... 10

2.1. GIXRD ... 10

2.2. XPS analysis of the as-prepared materials ... 11

2.3. Electrode activity ... 13

2.4. Chronoamperometry measurements ... 14

2.5. Electrode stability ... 16

2.6. In situ Raman measurements ... 17

2.7. Pulsed voltammetry ... 20

2.8. Double layer capacitance ... 21

2.9. Oxidation of the Cannizzaro products ... 22

2.10. Continuous flow cell experiments ... 23

2.11. Ag/GDE characterization ... 26

3. Estimation of Syngas and FDCA production ... 29

4. References ... 37

1. Instruments and experimental procedures

1.1. Chemicals and materials

All chemicals were used as received, without further purification unless otherwise specified.

Chemicals: 5-hydroxymethylfurfural (HMF, stored at 3 °C; AVA-Chemicals, Switzerland, 98%), 2,5-furandicarboxylic acid (FDCA; Alfa Aesar, 98%), diformylfuran (DFF; Sigma-Aldrich, 97%), 5-hydroxymethyl-2-furancarboxylic acid (HMFCA; BLDpharm, 99%), 5-formyl-2-furancarboxylic acid (FFCA; TCI, 98%), dihydroxymethylfuran (DHMF; BLDpharm, 98%), iron(III) chloride hexahydrate (reagent grade, ≥ 98%, Sigma-Aldrich), cobalt(II) chloride hexyhydrate (Sigma-Aldrich, 98% ACS), 35 wt% H_2O_2 (J. T. Baker), potassium hydroxide (KOH, reagent pellet; VWR Chemicals ≥ 85%), Ni foam (Recemat BV, Netherlands; Ni-4753), Ni sheets (MetallEhrnsberger, Germany, LC-Ni99, >99.2%) and perfluorinated membrane made from Nafion™ 117 (Sigma-Aldrich). Silver nanopowder (< 100 nm particle size, 99.5% trace metals basis); Sorbitol (99%), $KHCO_3$ (99.5%) and KOH (99.9%) were purchased from Sigma Aldrich and used as received. PTFE-treated carbon papers with a microporous layer (Freudenberg H23C6) were obtained from Fuel Cell Store and afterwards cut into desired dimensions (3 cm x 3.4 cm) using a razor blade. The entire micro flow cell assembly, and a leakless Ag/AgCl micro reference electrode were sourced from Electrocell (microflow cell). Bipolar membranes (BPMs) and Sustainion® XA-9 Alkaline Ionomer (5 wt%, in a mixture of lower aliphatic alcohols and water) were purchased from Dioxide Materials. BPMs were stored in 1 M NaCl solution and placed in a closed container.

Before synthesis, all flasks were washed with HCl to remove any metal residue, followed by rinsing with water and acetone. The flasks were then dried at 80 °C overnight. Milli-Q water (18.2 MΩ) was used for all synthesis and catalysis experiments.

1.2. Preparation of electrodes

Synthesis of Fe-modified Ni substrates:

The synthesis procedure for preparing transition metal-modified nickel foams (NF) or Ni sheets (Ni) was adapted from a previously published protocol.[1] Prior to the modification process, the Ni substrates were cut into slices (1 cm × 3 cm) and cleaned by ultrasonication sequentially in 3 M hydrochloric acid, acetone, ethanol, and ultrapure water for 15 min each. Meanwhile, a solution containing $FeCl_3 \cdot 6H_2O$ (2.5 mmol) in 15 mL of water was prepared in a 50 mL beaker. Next, 10 mL of an H_2O_2 solution was added to reach a total volume of 25 mL with an initial H_2O_2 concentration of 5 wt%. After 4 min of reaction, the cleaned Ni substrate was introduced into the mixture. Following 1 minute of treatment, the solution was decanted, and the substrate was rinsed with MQ water. Finally, the modified substrate was placed in a plastic Petri dish and dried at 60 °C in an oven for 24 hours.

Synthesis of FeCo-modified Ni substrates:

The synthesis of FeCo-modified Ni substrates with a defined Fe/Co ratio was performed identically to the previously described method. However, in addition to the 2.5 mmol of $FeCl_3$, which was kept constant for all Fe-modified samples, a variable amount of $CoCl_2$ was added to prepare a 15 mL solution with the desired Fe/Co ratio. Similarly, an H_2O_2 solution was added to reach a total volume of 25 mL with an initial H_2O_2 concentration of 5 wt%. After 4 min of reaction time, the cleaned Ni substrate was introduced and removed after 1 minute of treatment. Subsequently, the solution was decanted, and the substrate was rinsed with MQ water. Finally, the modified substrate was placed in a plastic Petri dish and dried at 60 °C in an oven for 24 hours.

Synthesis of Co-modified Ni substrates:

The synthesis of Co-modified Ni substrates was performed using the same protocol as that for Fe-modified Ni substrates. However, the 2.5 mmol of $FeCl_3$ was replaced with 2.5 mmol of $CoCl_2$.

H$_2$O$_2$ treated Ni substrates:

The synthesis of H_2O_2-treated Ni substrates was performed using the same protocol as that for Fe-modified Ni substrates. However, the Ni substrates were added to a pure 5 wt% H_2O_2 solution in the absence of any transition metal salts.

Gas diffusion electrode preparation:

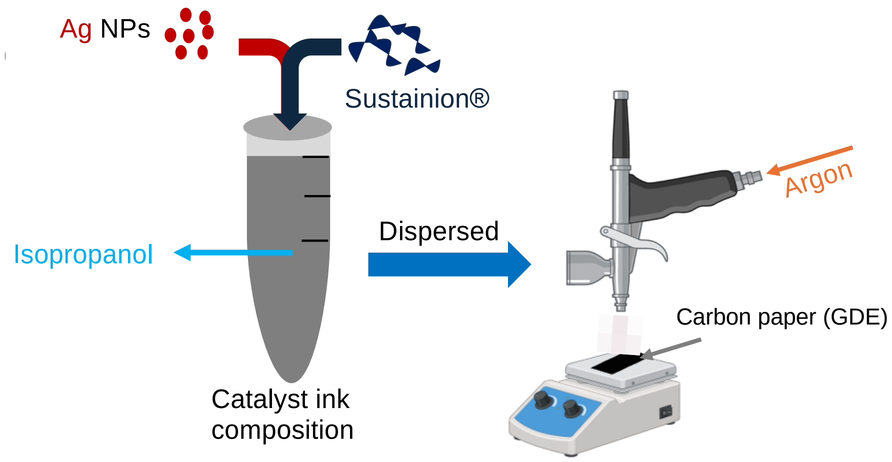

Figure S1: *Schematic illustration of the cathode silver gas diffusion electrode (GDE) preparation by airbrushing.*

A silver (Ag) catalyst ink using commercial Ag powder was prepared by dissolving 10 mg of catalyst and 100 µL of Sustainion® XA-9 alkaline ionomer in 1 mL of isopropyl alcohol, followed by ultrasonication for 90 min. Subsequently, the catalyst ink was applied using manual airbrushing onto a 10.2 cm^2 hydrophobic carbon paper with a microporous layer (Freudenberg paper H23C6), with an argon pressure of 1.5 bar in the airbrush (Figure S1). To facilitate solvent evaporation during manual airbrushing, the hotplate beneath the GDE was set to 150 °C. The carbon paper was weighed before and after catalyst deposition, yielding in a catalyst mass loading of approximately 0.7 mg cm^{-2} after drying.

The detailed characterization of the Ag/GDE electrode is described in Chapter 2.11.

1.3. Electrode characterization

Inductively Coupled Plasma-Optical Emission Spectrometry (ICP-OES):

Measurements were conducted using a Spectro Green FMX 46 ICP-OES. Nickel foams were dissolved in aqua regia at elevated temperatures. The resulting solution was then diluted with MQ water prior to the measurement. The signals were quantified using a multi-element standard obtained from Bernd Kraft.

X-Ray photoelectron spectroscopy (XPS)

X-ray photoelectron spectroscopy (XPS) was performed using a custom spectrometer from SPECS GmbH, equipped with a Phoibos 150 hemispherical energy analyzer and a 1D-DLD detector. The Mg X-ray source (E = 1253.64 eV) was operated at 15 kV and 200 W. For high-resolution scans, the pass energy was set to 20 eV, while for survey scans, it was set to 50 eV. The medium area mode was used as the lens mode. During the experiment, the base pressure in the analysis chamber was maintained at 3×10^{-9} mbar. Unless otherwise stated, spectra were referenced to the C 1s peak at 284.8 eV to correct for charging effects.

Grazing incidence X-ray diffraction (GIXRD)

The grazing incidence X-ray diffraction (GIXRD) data for qualitative phase analysis were collected using a Rigaku SmartLab diffractometer equipped with a rotating anode (9 kW, 45 kV, 200 mA)

operating in Bragg-Brentano geometry ($CuK\alpha_{1,2}$: 1.541862 Å). Data acquisition was performed using a HyPix-3000 multi-dimensional detector in 0D and XRF modes. A parabolic multilayer mirror was used to generate a parallel beam. Samples were placed on a glass sample holder, and data were collected continuously over a 2θ range of 25 – 85° with a step size of 0.04° and a scan speed of 1° min^{-1}. For each sample, four scans were recorded and summed post-acquisition. The take-off angle of the X-ray tube was set to 1.1°, and the incident slit opening was 0.09 mm, with a 5 mm mask.

<u>Scanning Electron Microscopy (SEM) CEST:</u>

Electron microscopy was conducted using a Zeiss Sigma EDVP scanning electron microscope (SEM), equipped with an Ametek EDAX analyzer for energy-dispersive X-ray spectroscopy (EDX) analysis.

<u>X-ray photoelectron spectroscopy (XPS) CEST:</u>

Raman measurements were conducted using an Invia Renishaw Raman microscope, equipped with a 532 nm laser excitation wavelength, an 1800 l/mm grating, and a 50× objective lens (Leica). The measurements were performed in a customized in situ electrochemical flow cell.[2] A Pt wire and a hydrogen electrode (HydroFlex, Gaskatel) were used as the counter and reference electrodes, respectively. A Ni sheet, treated according to one of the procedures described above, was used as the working electrode. During the measurement, the 0.1 M KOH flux was controlled using a peristaltic pump with a flow rate of 12 mL min^{-1}. Measurements in the presence of HMF were conducted by adding HMF to the solvent reservoir, resulting in an HMF concentration of 10 mM. The Raman spectra were recorded in situ in chronoamperometric (CA) mode, with the potential held for 1 min at OCP, followed by a gradual potential bias increase from 1.0 V up to 1.6 V vs. RHE. Twenty consecutive scans were performed with a 3 s exposure time at 5 mW laser power under liquid environments to obtain the spectra.

1.4. In-situ Raman measurements:

Raman measurements were conducted using an Invia Renishaw Raman microscope equipped with a 532 nm laser excitation wavelength, an 1800 l/mm grating, and a 50× objective lens (Leica). The measurements were performed in a customized in situ electrochemical flow cell.[2] A Pt wire and a hydrogen electrode (HydroFlex, Gaskatel) were used as the counter and reference electrodes, respectively. A Ni sheet, treated according to one of the procedures described above, was used as the working electrode. During the measurement, the 0.1 M KOH flux was controlled using a peristaltic pump with a flow rate of 12 mL min^{-1}. Measurements in the presence of HMF were conducted by adding HMF to the solvent reservoir, resulting in an HMF concentration of 10 mM. The Raman spectra were recorded in situ in chronoamperometric (CA) mode, with the potential held for 1 min at OCP, followed by a gradual potential bias increase from 1.0 V up to 1.6 V vs. RHE. Twenty consecutive scans were performed with a 3 s exposure time at 5 mW laser power under liquid environments to obtain the spectra.

1.5. Electrochemical measurements in batch:

<u>Electrochemical measurements:</u>

Electrochemical measurements were performed at room temperature using a Gamry Interface 1010 B electrochemical workstation with a three-electrode system in an H-type cell, separated by a Nafion 117 membrane. A coiled Pt wire served as the counter electrode (CE), an Hg/HgO electrode (internal solution: 1 M KOH) as the reference electrode (RE), and Ni foam (1 cm × 1 cm) as the working electrode (WE). All reactions were conducted in 5 mL of the corresponding electrolyte, with organic substrates added exclusively to the anode compartment. Stirring was applied at 250 rpm in the cathode chamber and 500 rpm in the anode chamber. In a typical electrochemical experiment sequence, cyclic

voltammetry (CV) was performed at a scan rate of 100 mV/s from 0.00 V to 0.70 V vs. Hg/HgO for 20 cycles to ensure stable electrode performance. Linear sweep voltammetry (LSV) was then carried out at a scan rate of 5 mV/s to evaluate the electrode performance in the electrochemical reaction. The potential between the WE and RE was measured as the potential versus the Hg/HgO reference electrode, which was subsequently converted to the potential versus the reversible hydrogen electrode (RHE) using the following equation:

$$E_{RHE} = E^{0}_{Hg/HgO} + E_{Hg/HgO} + 0.0592 \cdot pH \quad (1.1)$$

$E^{0}_{Hg/HgO}$ here refers to the electrode potential for the Hg/HgO reference electrode (105 mV).[3]

Electrochemical double layer (C_{dl}) analysis was carried out by CV scans in the potential range of 0.95 V – 1.05 V vs. RHE with a scanning rate of 10, 20, 30, 40, 50, 60, 70, 80, 90, 100, 200, 300, 400, and 500 mV s^{-1}. The Δj/2 obtained at 0.10 V vs. RHE was plotted against the scan rate, and subsequently fitted by an allometric fit. The C_{dl} value was the slope of the fit.[4]

Pulsed voltammetry was performed by oxidation of the electrode at 1.45 V vs. RHE for 1 min followed by a 1 min long equilibration step at open circuit potential (OCP; here referring to open circuit conditions) and subsequent reductive treatment at 0.90 V vs. RHE for 2 min. Reduction in the presence of HMF was performed by the addition of HMF (50 mM) in the equilibration step.

Product analysis:

HPLC was used to quantitatively analyze the products of HMF oxidation and to calculate the corresponding Faradaic efficiencies (FE). Electrolyte samples were collected from the reaction cell, and immediately analyzed using an HPLC system, which consisted of a Shimadzu LC-2030 chromatograph, equipped with a 100 mm organic acid resin column (8.0 mm inner diameter), and a pre-column (40 mm organic acid resin, 8.0 mm i.d.).A 2 mM aqueous solution of trifluoroacetic acid was used as the mobile phase, at a flow rate of 1 mL min^{-1}, and a temperature of 40 °C. Organic substrates were detected using a UV detector set at 270 nm. External one-point calibration was applied to quantify HMF, HMFCA, DFF, FFCA, furoic acid, and FDCA. Yields γ and the FE were calculated using the following equations:

$$\gamma\ (\%) = \frac{c(product)}{c_0(educt)} \cdot 100\% \quad (1.2)$$

$$FE\ (\%) = \frac{c(product) \cdot N(e^-) \cdot F}{C} \cdot 100\% \quad (1.3)$$

$N(e^-)$ represents the number of transferred electrons required to oxidize the corresponding substrate to the product molecule (e.g., $N(e^-) = 6$ for HMF oxidation to FDCA), and C denotes the amount of induced charge. The Faraday constant F (96485.33 C mol^{-1}), serves as the proportionality factor.

Additional product analysis was performed using quantitative ^{1}H NMR measurements. NMR spectra were recorded using a Bruker 300 MHz AVIIIHD Nanobay NMR spectrometer. Spectra were generally measured at 300 K. ^{1}H chemical shifts are reported relative to DSS, using DMSO as the internal reference, as described by Babij *et al.*[5] All chemical shifts (δ) are given in ppm, coupling constants in Hz, and standard abbreviations are used. Quantitative data from reaction mixtures were extracted from ^{1}H-NMR spectra (spectral width = 2 ppm) using perfect echo excitation sculpting for water suppression before acquisition (Bruker pulse sequence: zgesgppe).[6] Typically, 8 FIDs containing 65,536 complex data points were averaged with a relaxation delay of 70 s between individual scans to ensure complete relaxation of all components. Before acquisition, the chemical shift of the water signal was automatically determined using an AU program (a modified version of Bruker's au_watersc with a shorter relaxation delay of 1 s). This value was then used as the central offset (O1) to ensure optimal water suppression. Although the selective pulse used in this sequence may suppress other signals, the quantitative results for the signals of interest were validated using reference samples. The concentration of the organics was determined using the following equation:

$$c_i = c(DMSO) \cdot \frac{I_i \cdot 6H}{I_{DMSO} \cdot N_i(H)} \cdot 100\% \quad (1.4)$$

I represents the measured integral, while *N(H)* denotes the number of protons associated with the signal. To prevent H/D exchange reactions in strongly alkaline media, a capillary filled with D_2O was used for locking and shimming. To slow down decomposition reactions, the electrolyte was diluted with MQ water. Since the signals of hydrogen atoms with ^1H chemical shifts close to the water signal are affected by the suppression sequence, these signals are neither labeled nor integrated, to avoid potential misinterpretation of the data. For exemplary measurements, the reader is referred to our previous study.[7]

1.6. Electrochemical measurements in flow:

Electrochemical measurements:

All electrochemical experiments were conducted within a microflow cell purchased from Electrocell, as illustrated in Figure 5, with electrode areas of 10.2 cm^2. The cell consists of three compartments: one for the anolyte, another for the catholyte, and a third for the gas compartment. All electrochemical experiments were conducted at room temperature and pressure using an Autolab PGSTAT302N potentiostat in three-electrode setup.

FeCo-modified Ni substrates were employed as the anode, while a leakless Ag/AgCl micro-reference electrode (saturated KCl) served as the reference electrode. Initially, a freshly prepared 0.5 M KOH solution was used as the anolyte, which was later replaced with a solution of the Cannizzaro products in 5 M KOH (initially 500 mM HMF before Cannizzaro conversion) for indirect HMFOR experiments. Different catholyte solutions, including freshly prepared 0.5 M $KHCO_3$ and 2 M phosphate buffers at pH values of pH 6, 7, and 8, were utilized for various experiments to optimize the syngas ratio. Both the anolyte and catholyte were circulated within the flow cell at a flow rate of 100 mL min^{-1} using a peristaltic pump. CO_2 was introduced at a flow rate of 60 sccm through the gas compartment on the cathode side. Ar, or no gas (for the Pt catalyst), was supplied when HER was used as the desired counter reaction. The catalyst GDE, bipolar membrane (BPM), and anode foam were assembled within the flow cell, and the electrolytes and gas were circulated for 30 min to saturate the system. Electrochemical stability was achieved through 15 CV scans ranging from 0 V to 0.5 V vs. Ag/AgCl reference electrode, at a scan rate of 10 mV/s. LSV measurements ranging from 0 V to 0.6 V vs. Ag/AgCl reference electrode were performed at a constant scan rate of 5 mV/s. An 85% IR compensation was carried out manually during data analysis. The potential between the WE and RE was measured as the potential versus the Ag/AgCl reference electrode, which was subsequently converted to the potential versus the RHE using the following equation:

$$E_{RHE} = E^0_{Ag/AgCl} + E_{Ag/AgCl} + 0.0592 \cdot pH \quad (1.5)$$

$E^0_{Ag/AgCl}$ here refers to the electrode potential for the Ag/AgCl reference electrode (198 mV at 25 °C).[8]

After introducing the Cannizzaro products in 5 M KOH on the anode side, chronopotentiometry (CP) experiments were performed at a constant current of 78.4 mA cm^{-2} for 6200 s for the HMFOR-CO_2RR coupled experiments. The CP duration was optimized to ensure complete conversion of the Cannizzaro products on the anode side. Meanwhile, gas products formed on the cathode side were analyzed by gas chromatography (GC) every 30 min.

Product analysis:

The collection of gaseous products was initiated after 300 s of electrolysis. Gaseous products were collected every 30 min from the outlet of the flow cell into Tedlar gas sampling bags. Samples were then injected into a Shimadzu Nexis GC-2030 gas chromatograph, using a gas-tight syringe. The GC utilized helium as the carrier gas, with a total flow rate of 35.4 mL min^{-1} under pressure flow control mode. The pressure was initially set at 226.8 kPa and held for 2.5 min; then increased at 15.2 kPa min^{-1} to 390.1 kPa, and held for 5.95 min, and finally increased at 11.2 kPa min^{-1} to 405.1 kPa, held for

5.42 min. The column used was a ShinCarbon ST Micropacked GC Column (100/120, 1 mm ID, 2 m). The column temperature was ramped from 50° C to 270° C. The temperature program started at 50 °C, held for 2.5 min. then increased at a rate of 10 °C min^{-1} to 250° C, followed by an increase at a rate of 9 °C min^{-1} to 270 °C, which was held for 8 min. Barrier Ionization Discharge (BID) detector was used with a detector temperature of 280 °C and a He discharge flow rate of 50 mL min^{-1}.

The FE of gaseous products were calculated using the following equations:

$$FE\ (\%) = \frac{moles(product) \cdot N(e^{-}) \cdot F}{C} \cdot 100\% \qquad (1.6)$$

Cumulative liquid products were analyzed after electrolysis, using a Shimadzu LCMS-2020 liquid chromatograph-mass spectrometer. The analysis was conducted with a Shim-pack SCR-102H ion exchange column (300 mm x 8.0 mm, 7 µm), maintained at 70° C. Detection was performed with a UV SPD-40 V detector, set at a wavelength of 220 nm. The mobile phase flow rate was maintained at 0.8 mL min^{-1}.

1.7. HMF conversion via Cannizzaro reaction

The conversion of HMF via the Cannizzaro reaction was investigated in a previous study.[7] We found that the highest yields of Cannizzaro products (DHMF and HMFCA) were obtained by increasing the concentrations of both KOH and HMF, while simultaneously lowering the reaction temperature to 0 °C. To achieve this, an aqueous 5 M KOH solution was prepared and cooled to approximately 0 °C in an ice bath. Meanwhile, the desired amount of HMF was weighed into a glass flask and placed in the ice bath. The precooled 5 M KOH solution was then added to the HMF under vigorous stirring, resulting in a 1 M HMF solution in 5 M KOH. The mixture was stirred at 0 °C overnight, leading to the conversion of HMF into DHMF and HMFCA via the Cannizzaro reaction. Product quantification was subsequently carried out using both HPLC and NMR spectroscopy. Electrolysis experiments with the Cannizzaro products were performed without further product separation. The resulting stock solution was simply diluted with 5 M KOH to obtain the desired concentration of the Cannizzaro products.

At CEST, 50 mL of 5 M KOH solution was cooled to approximately –5 °C. Subsequently, 3.151 g of HMF (corresponding to 0.5 M) was added to the cooled KOH solution. The reaction was allowed to proceed for 24 hours. The resulting Cannizzaro products, DHMF and HMFCA, were quantified by HPLC, and the total yields are reported in Table S1. This product’s solution was then directly used for electrolysis, without any further dilution.

Table S1 presents the yields from multiple conversion runs across two laboratories:

Table S1: *Yields obtained from the conversion of HMF to DHMF and HMFCA via the Cannizzaro reaction under highly alkaline conditions.*

Laboratory	Volume / mL	c_0(HMF) / mol L^{-1}	c_0(KOH) / mol L^{-1}	Yields[b] / %
MPI KOFO	20	1	5	85.4
MPI KOFO	20	1	5	83.8
MPI KOFO	20	1	5	84.2
MPI KOFO	10	1	5	88.3
MPI KOFO	10	1	5	86.5
MPI KOFO	10	1	5	90.6
CEST	50	0.5	5	74.8
CEST	50	0.5	5	82.3
CEST	50	0.5	5	79.8
CEST	50	0.5	5	84.9
CEST	50	0.5	5	82.8

[a]*Institut abbreviation:*

MPI KOFO: Max-Planck-Institut für Kohlenforschung; Mülheim; Germany

CEST: Center for Electrochemistry and Surface Technology; Wiener Neustadt; Austria

[b]*Yields refer to the combined yields of HMFCA and DHMF based on the initial HMF input.*

2. Results

2.1. GIXRD

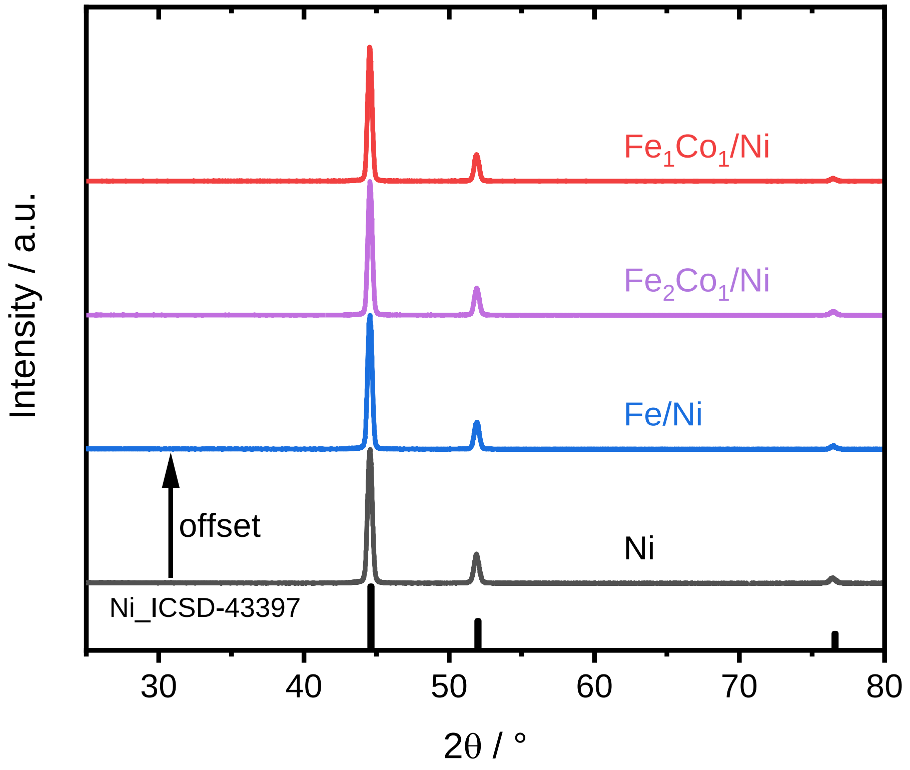

Figure S2: *Grazing incidence diffraction pattern of the H_2O_2 treated Ni electrodes.*

2.2. XPS analysis of the as-prepared materials

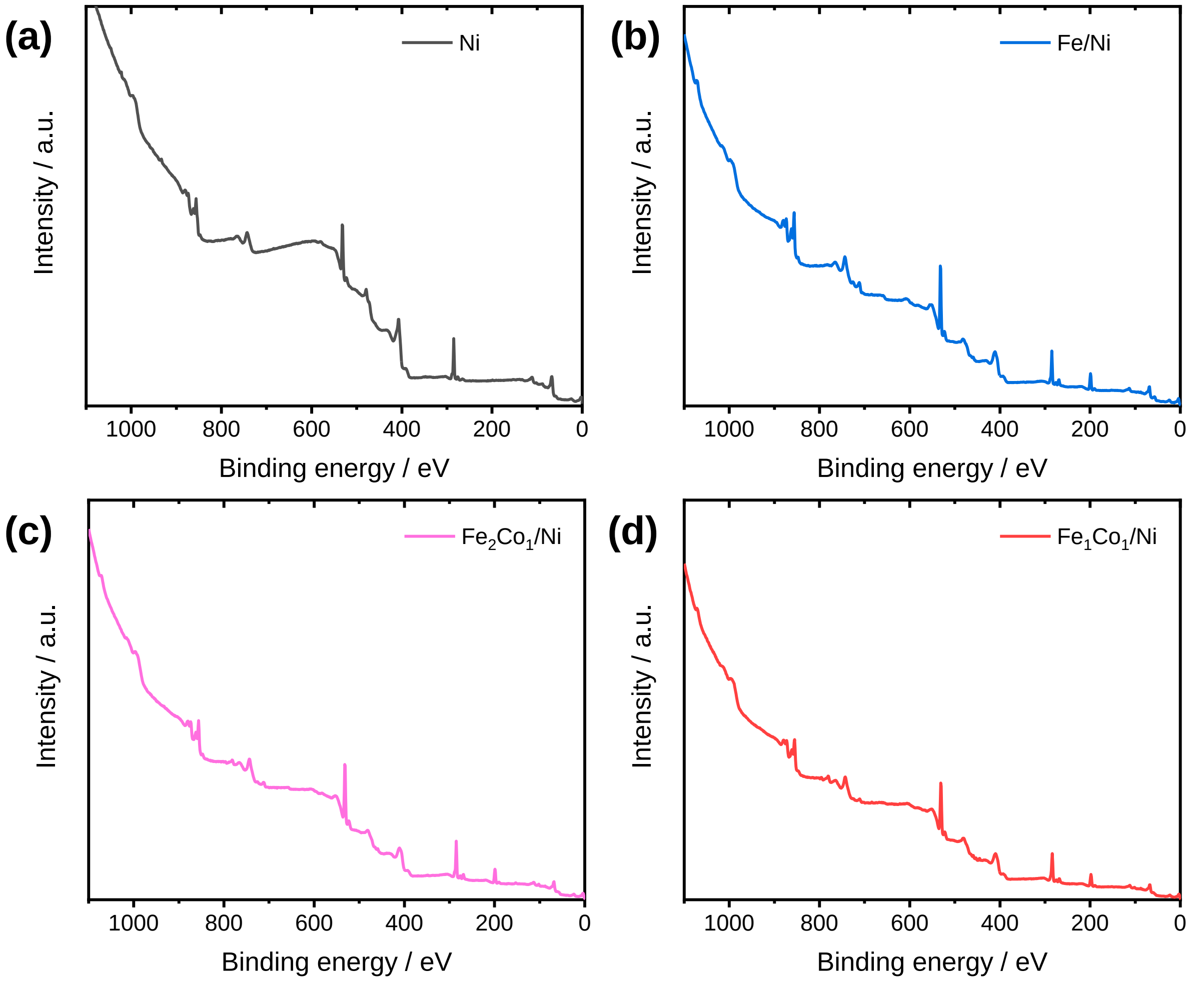

Figure S3: *XPS spectra for the different Ni electrodes synthesized by treatment of a Ni sheet with a mixture Fe and H_2O_2. For the Co containing samples, $CoCl_2$ was added into the synthesis procedure. (a) H_2O_2 treated Ni substrate ,(b) Fe/NF, (c) Fe_2Co_1/NF, (d) Fe_1Co_1/NF.*

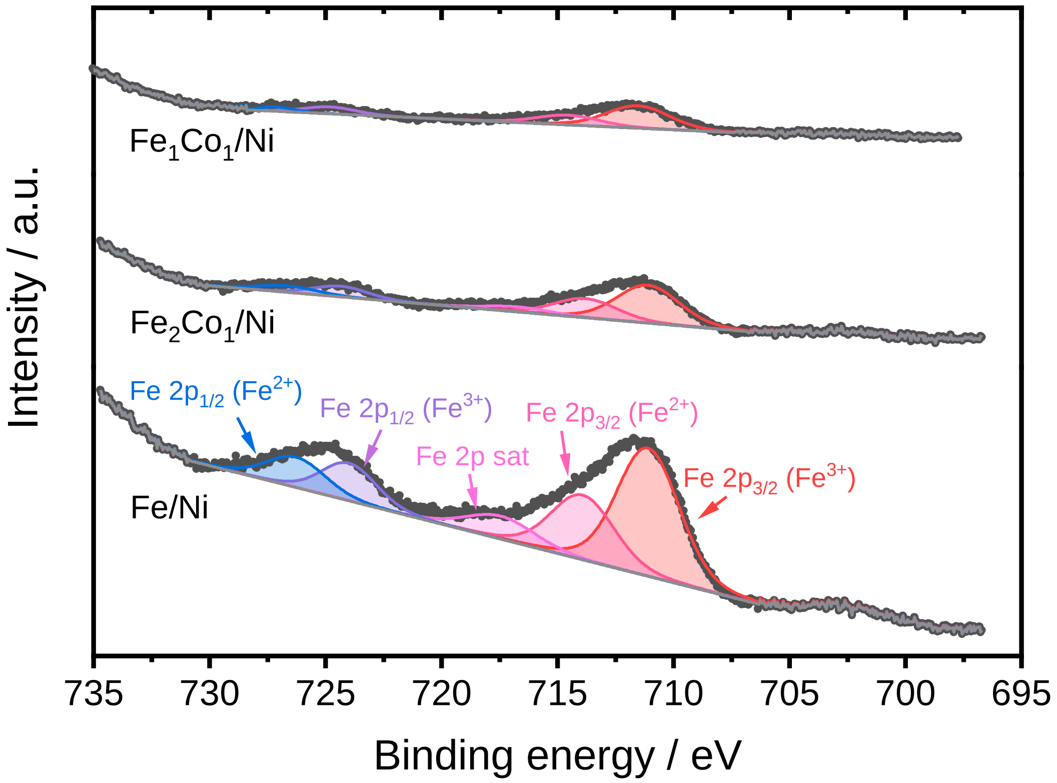

Figure S4: *Fe 2p XPS spectra for the different Ni electrodes synthesized by treatment of a Ni sheet with a mixture Fe and* H_2O_2*. For the Co containing samples,* $CoCl_2$ *was added into the synthesis procedure.*

2.3. Electrode activity

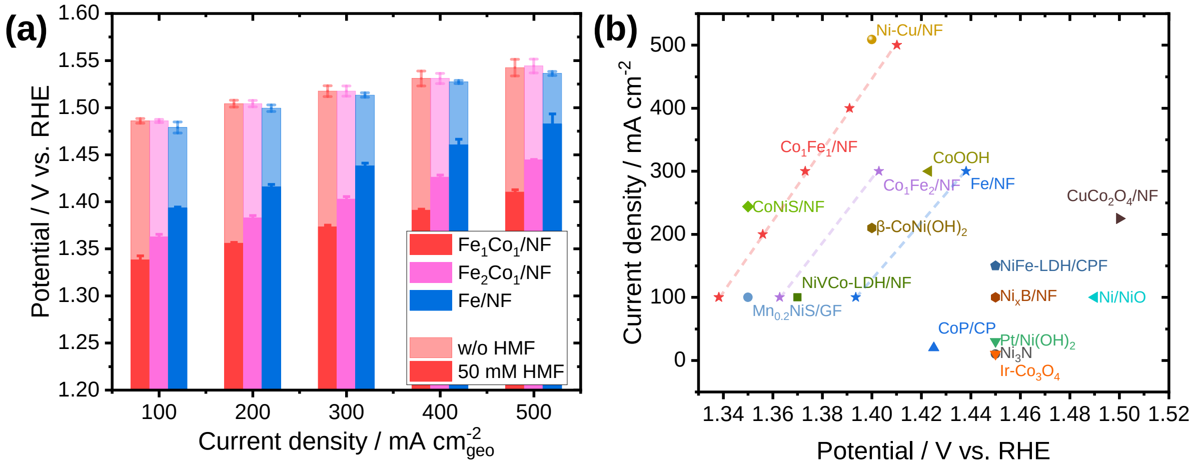

Figure S5: *(a) Comparison of the average required potential for different electrode materials to reach a defined current density in the presence and absence of 50 mM HMF in 1 M KOH. The performance was evaluated by LSV measurements at a scan rate of 5 mV s^{-1}, without correction for the uncompensated resistance. Average values and standard deviations were obtained from measurements on at least three individual electrodes. (b) Average performance of the as-prepared electrodes (star) compared to a selection of other electrodes reported in the literature, tested under comparable conditions (see table S2). Dotted lines are used for visual guidance.*

Table S2: *Data for the comparison of different electrode materials in the HMF electrooxidation.*

Electrode	c(KOH) / mol	c(HMF) / mmol	iR-correction	Potential / V vs. RHE	Current density / mA cm^{-2}	Reference
Fe/NF	1.0	50	no	1.44	300	-
Fe_2Co_1/NF	1.0	50	no	1.40	300	-
Fe_1Co_1/NF	1.0	50	no	1.37	300	-
CoNiS/NF	1.0	50	no	1.35	244	[9]
$Mn_{0.2}NiS/GF$	1.0	100	no	1.35	100	[10]
Ni-Cu/NF	1.0	50	Yes; 100%	1.40	509	[11]
CoOOH	1.0	50	Yes	1.423	300	[12]
CoP/CP	1.0	50	Yes	1.425	20	[13]
$Ir-Co_3O_4$	1.0	50	Yes	1.45	10	[14]
Ni/NiO	1.0	50	Yes	1.49	100	[15]
$Pt/Ni(OH)_2$	1.0	50	Yes; 95%	1.45	30	[16]
Ni_3N	1.0	50	Yes	1.45	10	[17]
Ni_xB	1.0	10	Yes	1.45	100	[18]
NiVCo-LDH/NF	1.0	10	no	1.37	100	[19]
NiFe-LDH/CPF	1.0	10	no	1.45	150	[20]
$CuCo_2O_4/NF$	1.0	50		1.5	225	[21]
β-$CoNi(OH)_2$	1.0	50	Yes, 90%	1.4	210	[22]

2.4. Chronoamperometry measurements

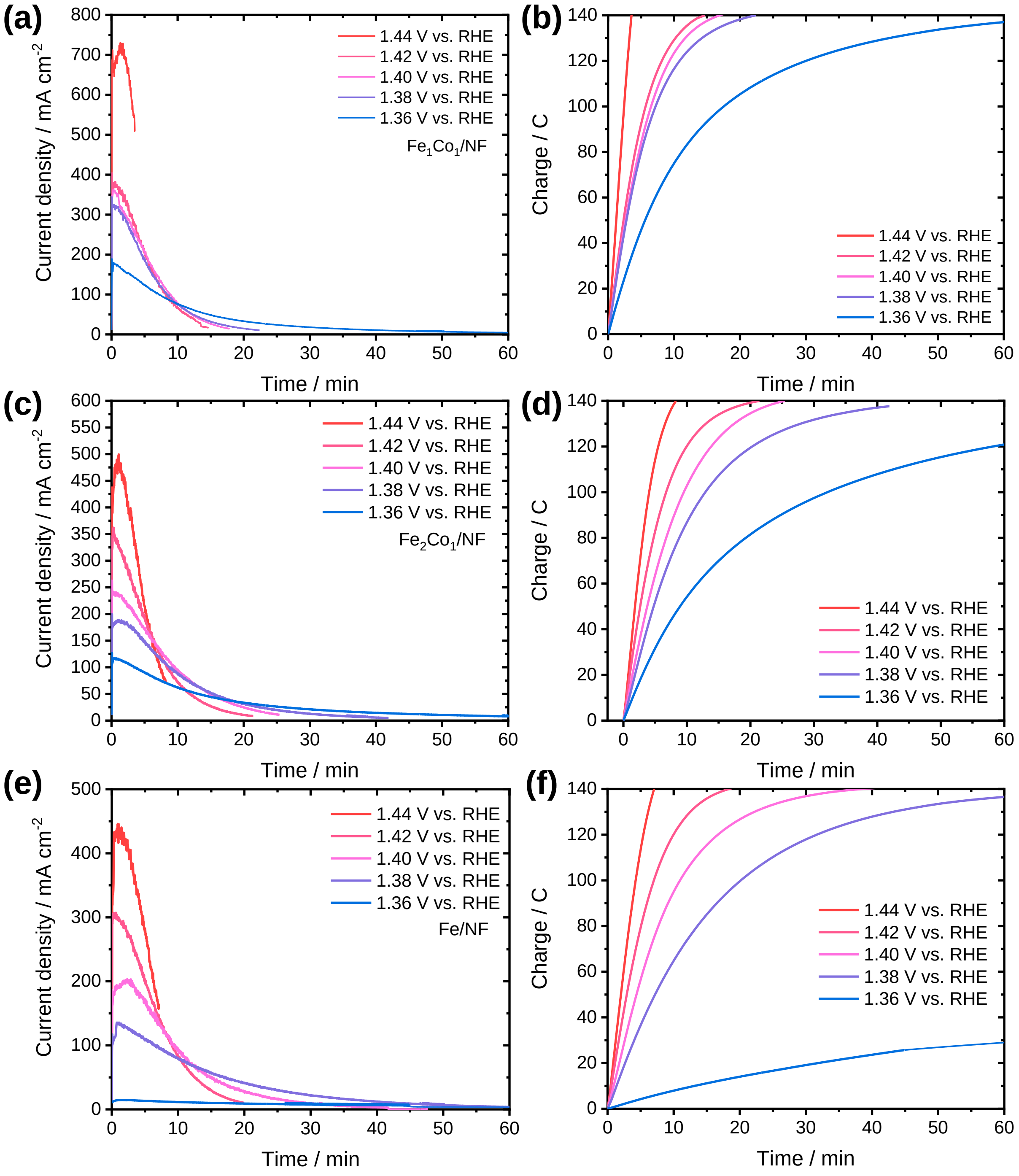

Figure S6: *Chronoamperometry measurements at different potentials. (a – b) Fe_1Co_1/NF, (c – d) Fe_2Co_1/NF, (e – f) Fe/NF.*

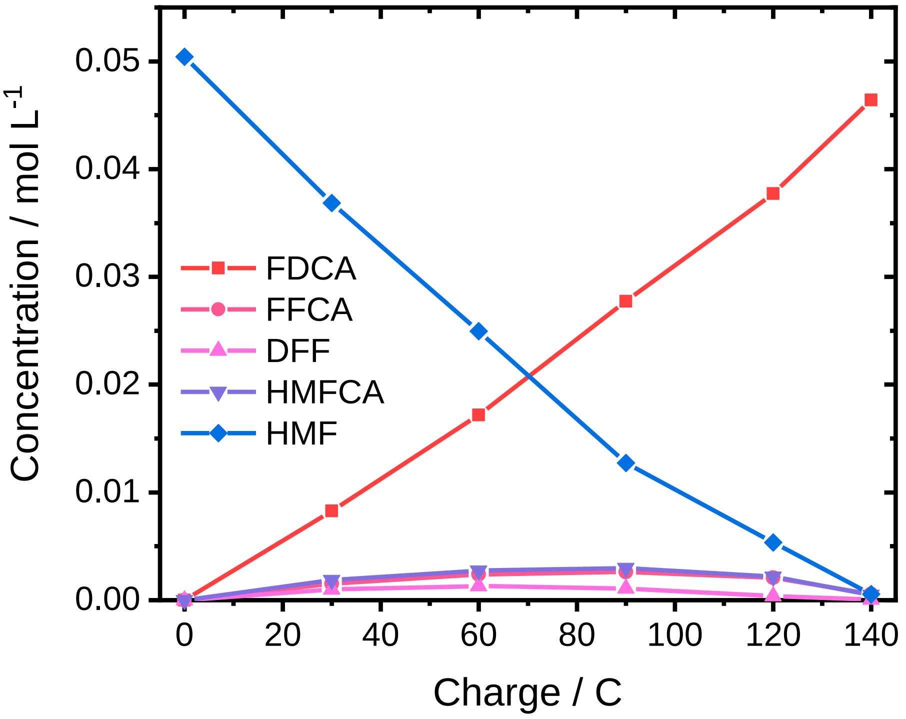

***Figure S7**: Product formation using the Fe_2Co_1/NF electrode in 1 M KOH in the presence of 50 mM HMF. Chronoamperometry measurements were performed at 1.40 V vs. RHE.*

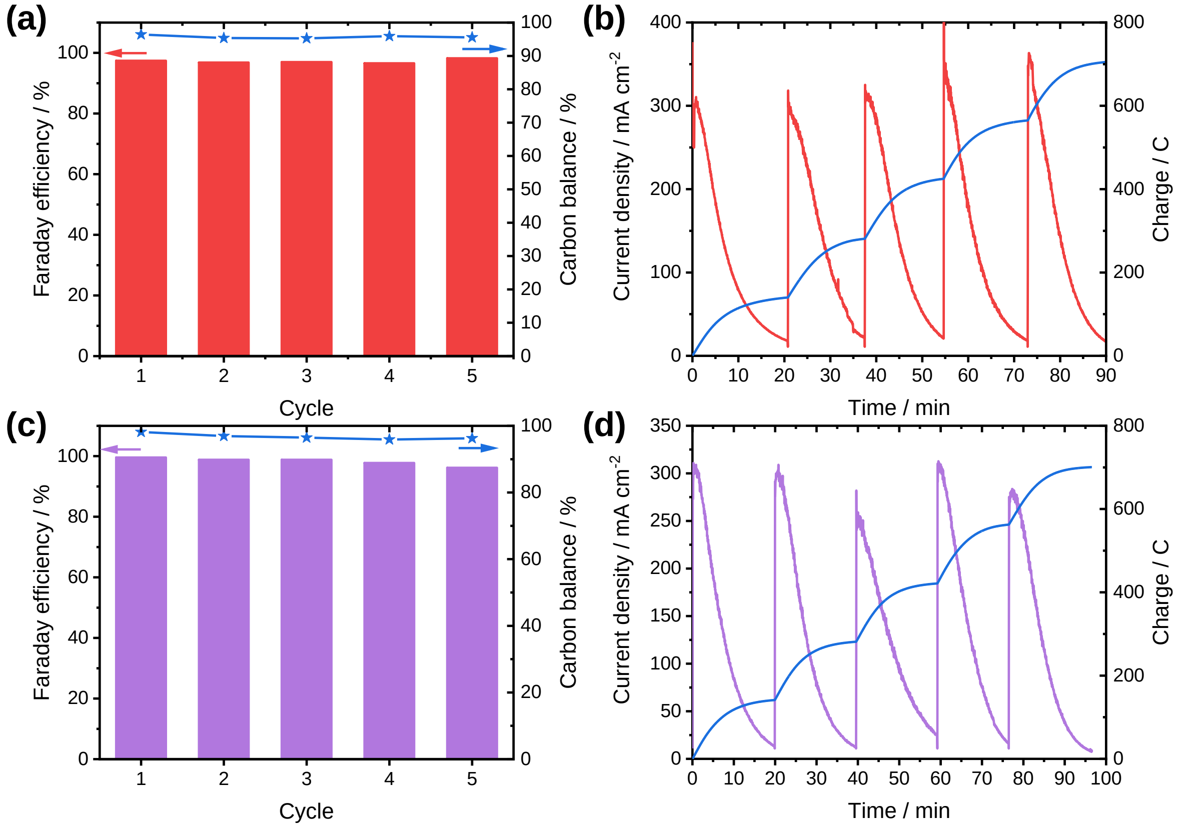

***Figure S8:** Chronoamperometry measurements performed at 1.40 V vs. RHE in 1 M KOH in the presence of 50 mM of HMF. After each electrolysis cycle, the electrolyte was fully exchanged. (a – b) Fe_1Co_1/NF, (c – d) Fe_2Co_1/NF.*

2.5. Electrode stability

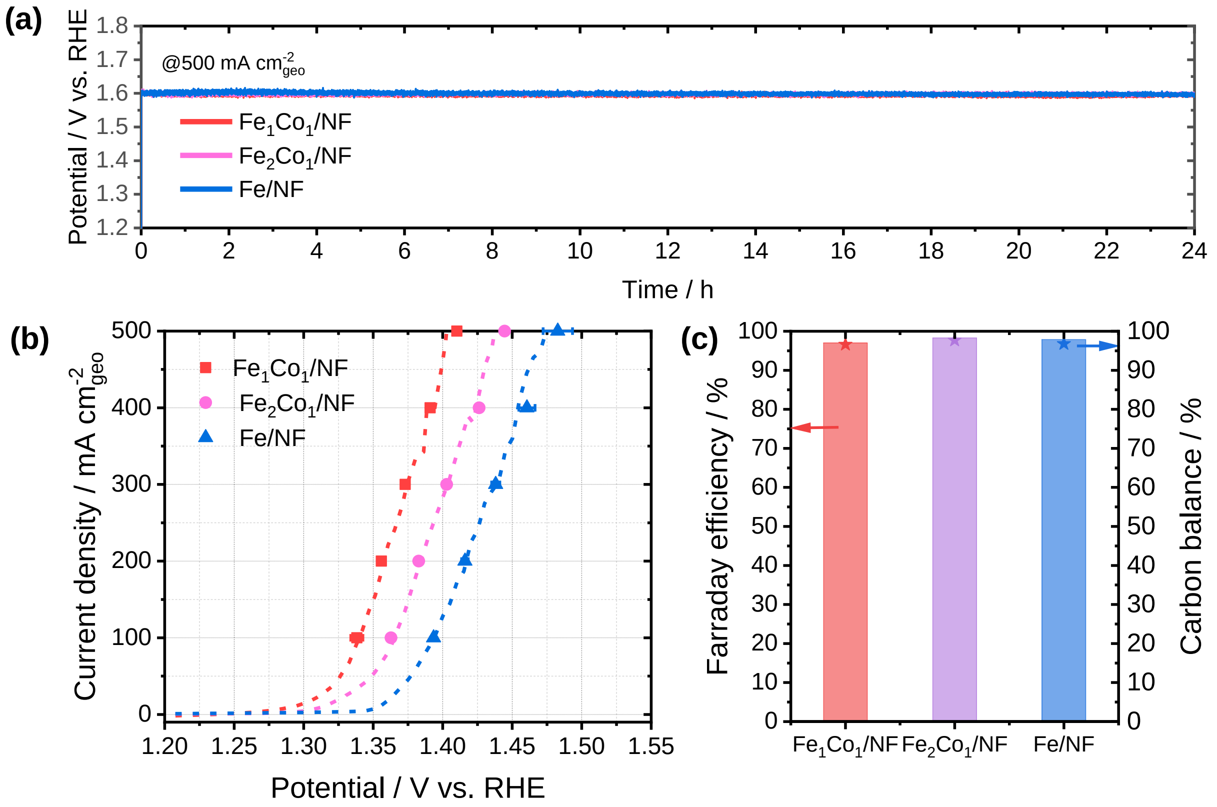

Figure S9: *(a) Chronopotentiometry measurements in 1 M KOH at 30 °C. (b) Subsequent LSV measurements (without iR correction) in the presence of 50 mM HMF in 1 M KOH. For comparison, dots and scatter display the average activity of the as-prepared material. Scan rate: 5 mV s⁻¹. (c) FE for FDCA and CB measured in chronoamperometry measurements at 1.40 V vs. RHE using the spent electrodes in the presence of 50 mM HMF in 1 M KOH.*

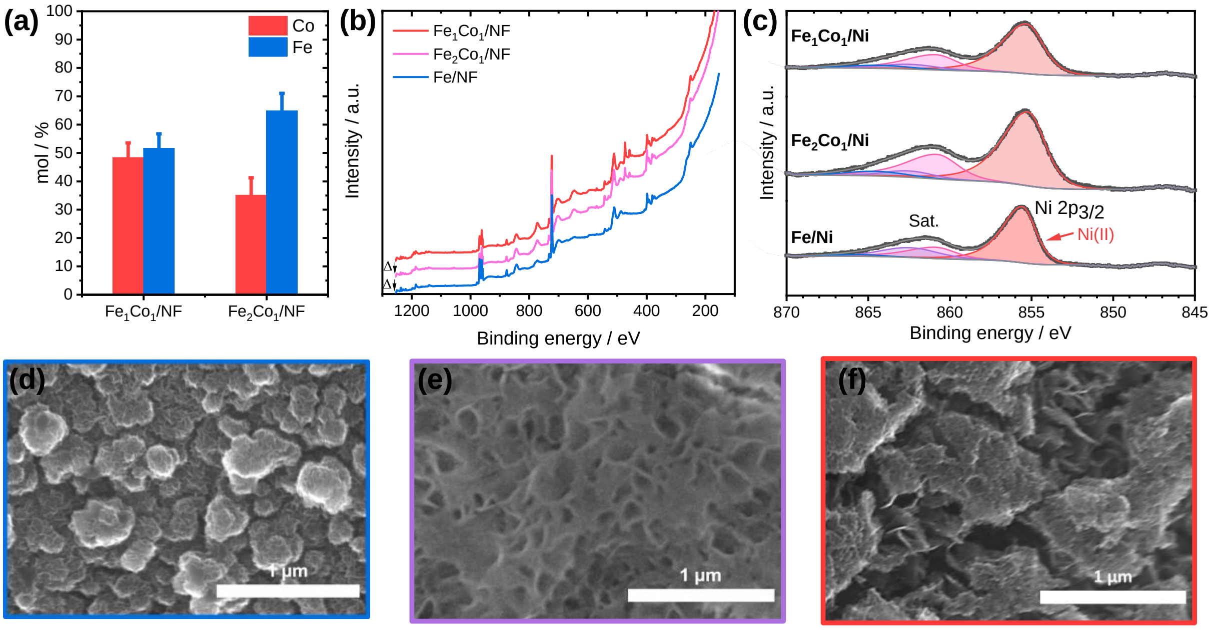

Figure S10: *Characterization o the spent electrode materials. (a) ICP-OES analysis. (b) Measured XPS spectra, (c) Ni 2p XPS spectra showing a fully oxidized Ni surface. (d) SEM image of the Fe/NF electrode. (e) SEM image of the Fe_2Co_1/NF electrode, (f) SEM image of the Fe_1Co_1/NF electrode.*

2.6. In situ Raman measurements

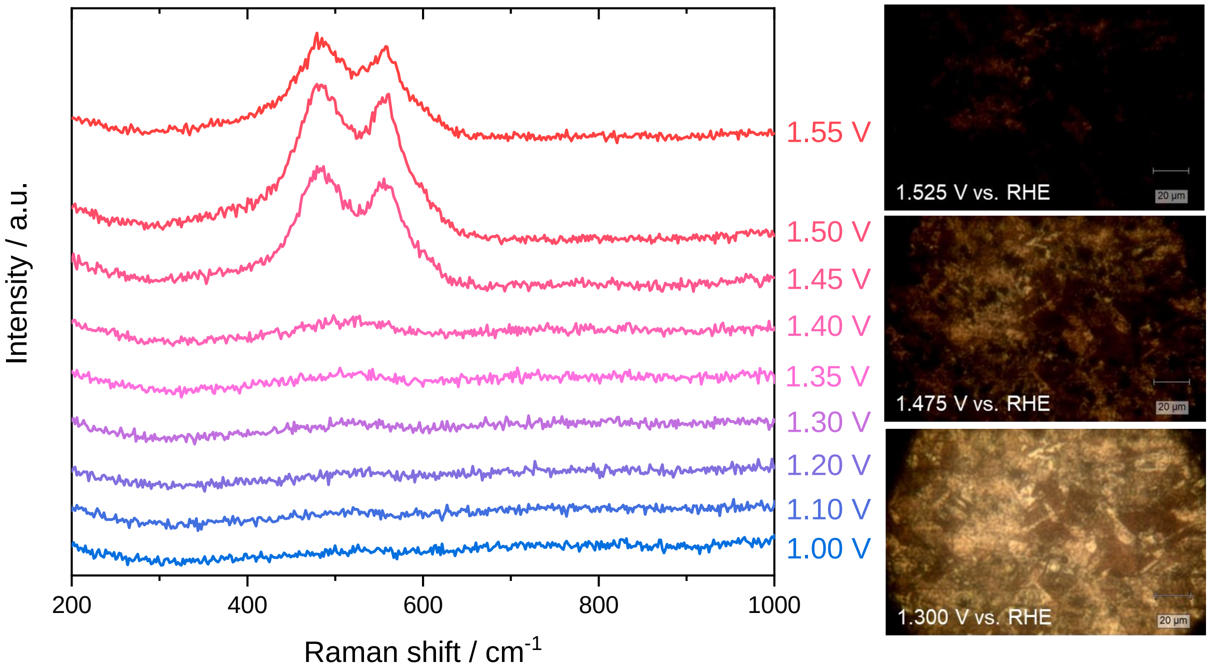

Figure S11: *In situ Raman measurements of Fe/NF under increasing potentials in 0.1M KOH. Images are taken by the microscope after surface oxidation, showing an increasingly darkened surface of the electrode, likely originating from the formation of NiOOH species, as evidenced by the emerging Raman bands.*

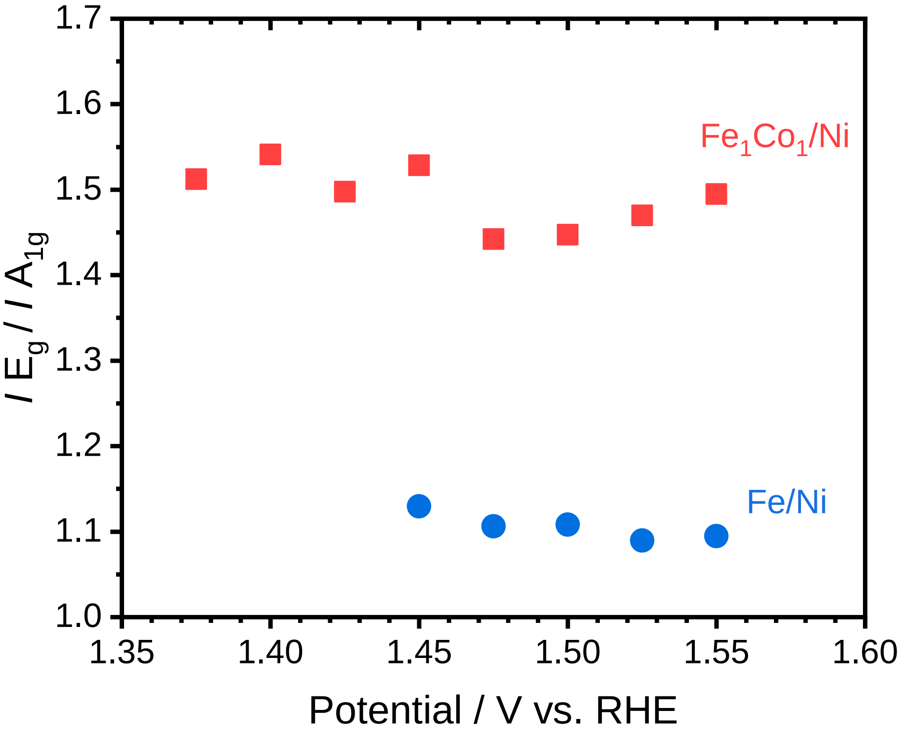

Figure S12: *Comparison of the E_g and A_{1g} band intensity ratio in dependence on the used electrode and applied potential. Fe/Ni (blue), Fe_1Co_1/Ni (red).*

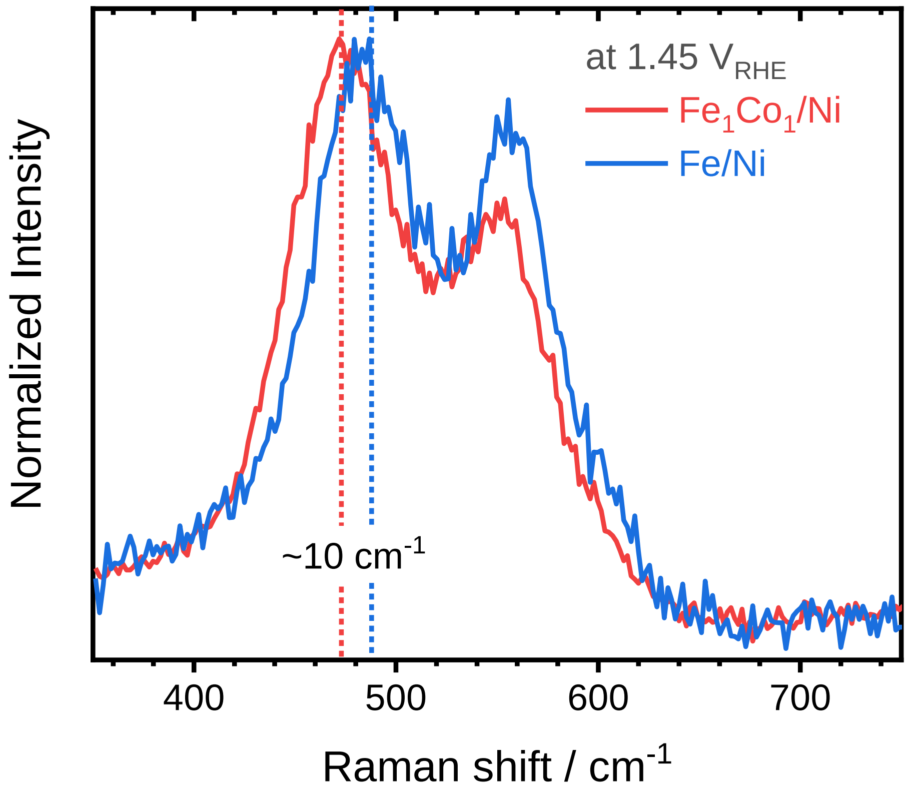

Figure S13: *Comparison of the peak positions of the Raman bands associated with the in-situ formed NiOOH species at 1.45 V vs. RHE, depending on the electrode used.*

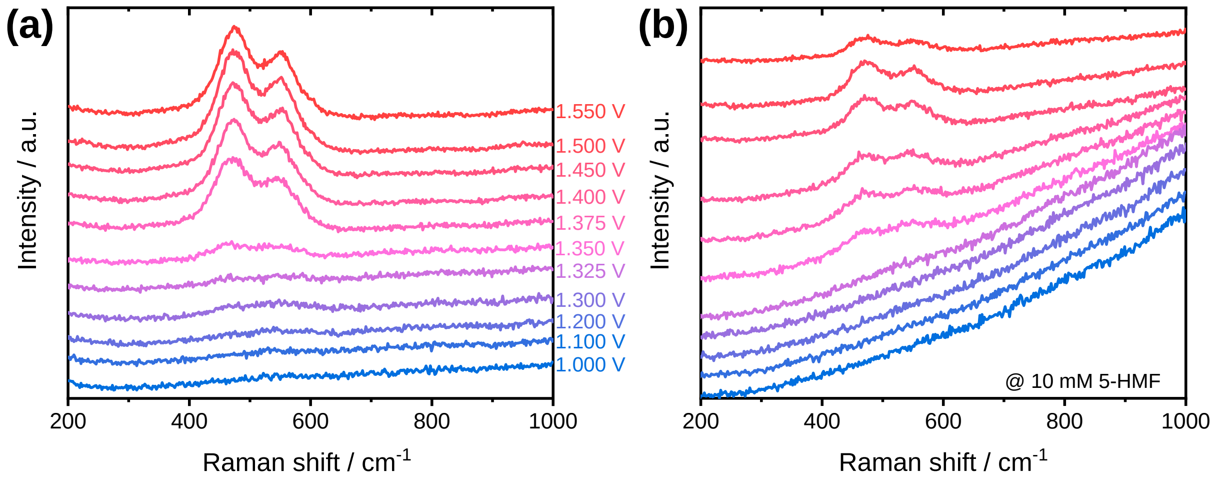

Figure S14: *In-situ Raman measurements of Fe_1Co_1/NF under increasing potentials in 0.1M KOH. (a) Raman spectra in the absence of HMF. (b) Raman spectra in the presence of 10 mM HMF.*

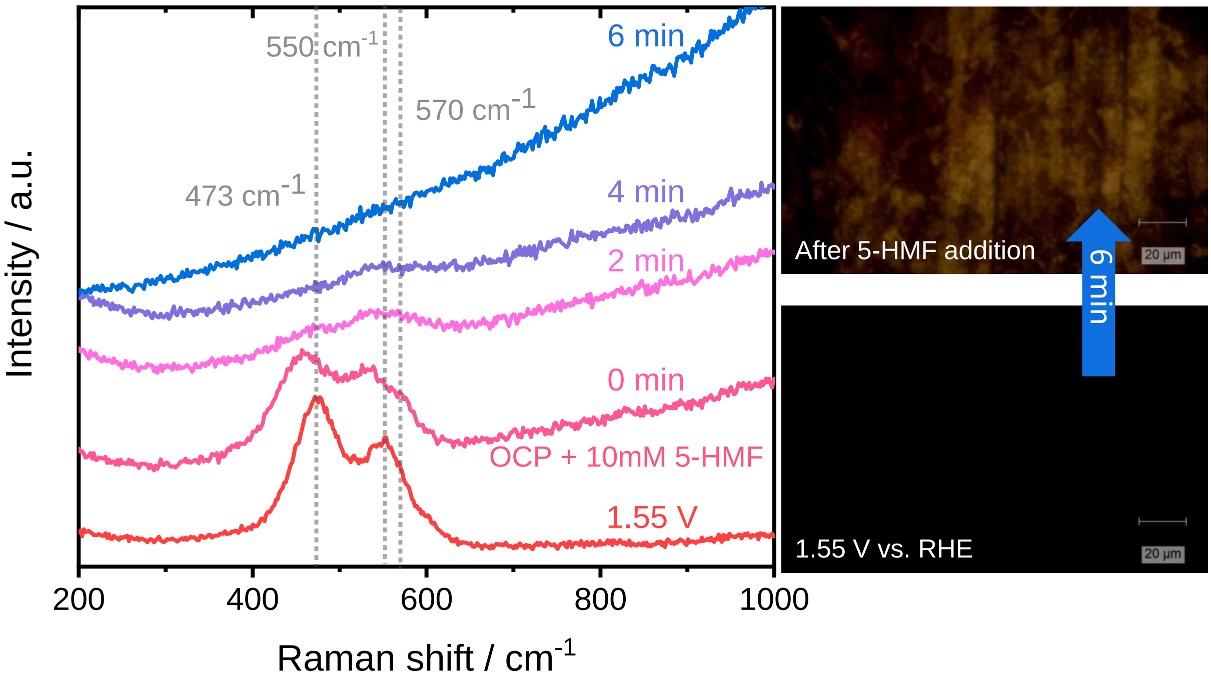

Figure S15: *In-situ Raman spectra of Fe_1Co_1/NF recorded in 0.1 M KOH. The initial spectrum shows the formation of a NiOOH species in the absence of HMF at 1.55 V vs RHE. Subsequently, HMF (10 mM) was added to the electrolyte and Raman spectra were collected every 2 min at OCP. Within a 6-minute timeframe, the intensity of the Raman bands gradually decreased. Microscope images taken after surface oxidation and after HMF addition showed that the electrode surface changed from deep black to a brownish appearance, further demonstrating the reduction of the initially present NiOOH phase by a spontaneous reaction with the added HMF.*

2.7. Pulsed voltammetry

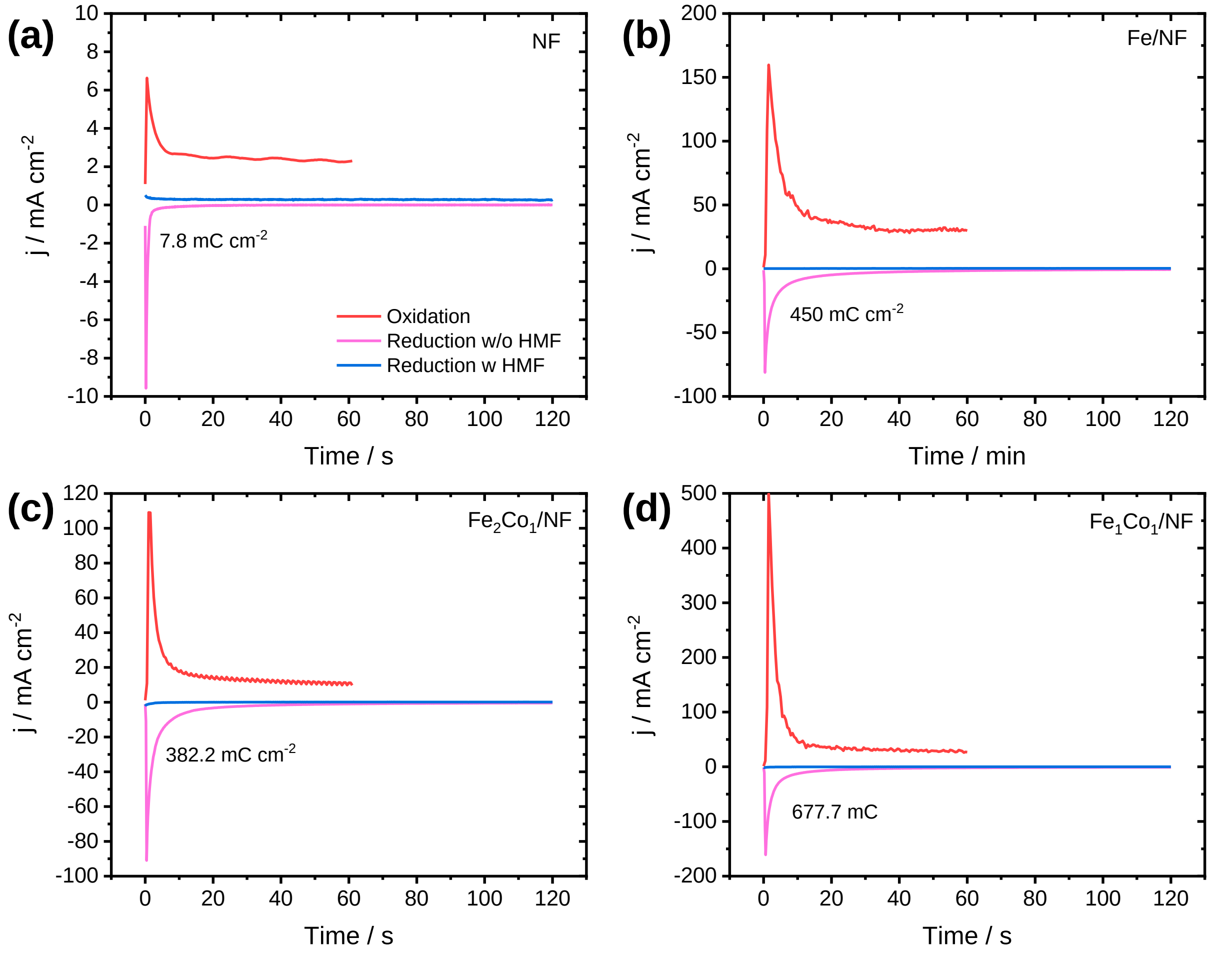

Figure S16: *Exemplary pulsed voltammetry results of different tested electrodes in 1 M KOH. (a) H_2O_2 treated NF, (b) Fe/NF, (c) Fe_2Co_1/NF, and (d) Fe_1Co_1/NF First the electrode is oxidized at an oxidizing potential of 1.45 V vs. RHE (red) and is subsequently, after an equilibration step at OCP, presented with a reducing potential of 0.9 V vs. RHE. Reduction was performed either in presence of 50 mM HMF (blue) or absence of HMF (violet).*

2.8. Double layer capacitance

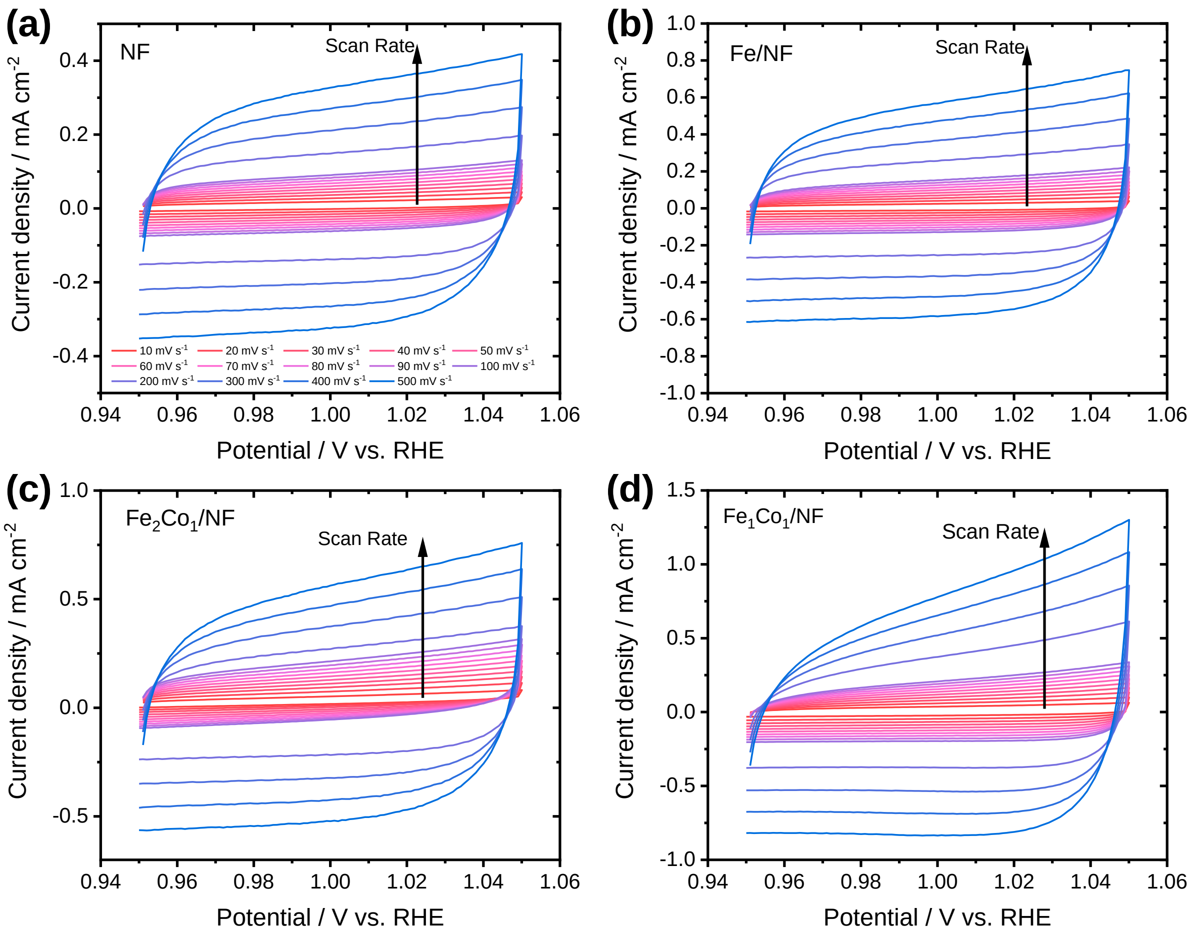

Figure S17: *C_{dl} measurements of different electrodes in 1 M KOH. (a) NF, (b) Fe/NF, (c) Fe_2Co_1/NF, (d) Fe_1Co_1/NF. Measurements were performed at 0.95 V to 1.05 V vs. RHE with 14 individual scan rates from 10 mV s^{-1} to 500 mV s^{-1}.*

2.9. Oxidation of the Cannizzaro products

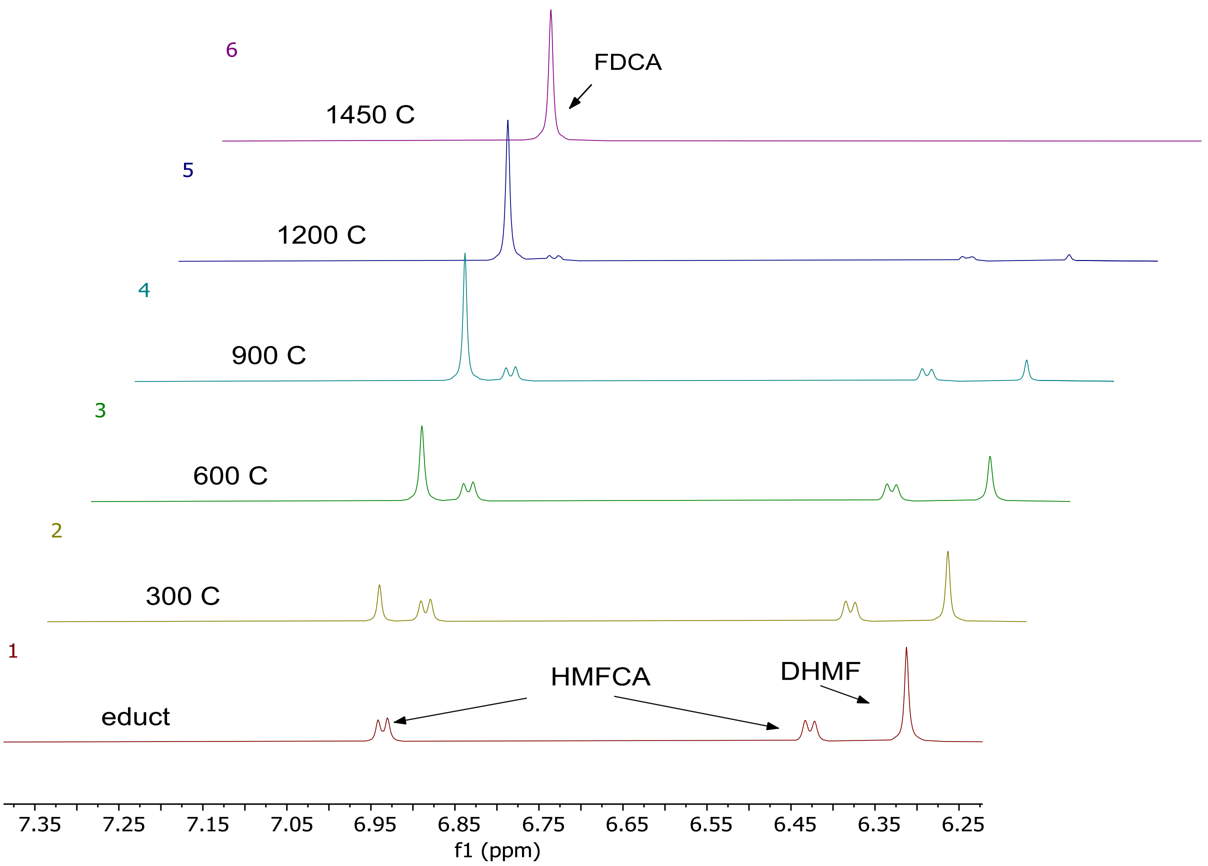

Figure S18: *^{1}H-NMR measurements used to visualize the educt conversion and FDCA formation during the electrolysis of a 500 mM concentration of the Cannizzaro products of HMF (DHMF and HMFCA) using the* Fe_1Co_1*/NF electrode in 5 M KOH.*

2.10. Continuous flow cell experiments

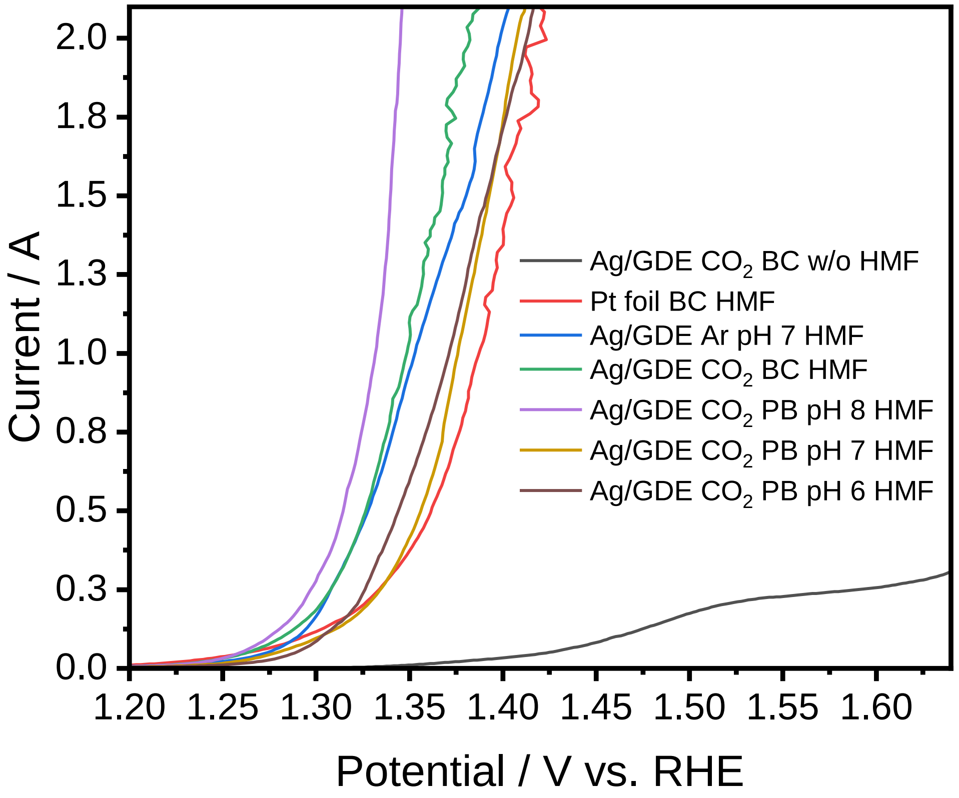

Figure S19: *Linear sweep voltammetry (LSV) results for the Fe_1Co_1/NF electrode in 5 M KOH, recorded in the presence and absence of 500 mM Cannizzaro products (based on initial HMF input before Cannizzaro conversion). Measurements were conducted at a scan rate of 5 mV s^{-1}. 85% manual iR-corrected was applied. The solution pH, calculated based on the KOH concentration, was estimated to be 14.7. All potentials were initially measured versus the Ag/AgCl electrode.*

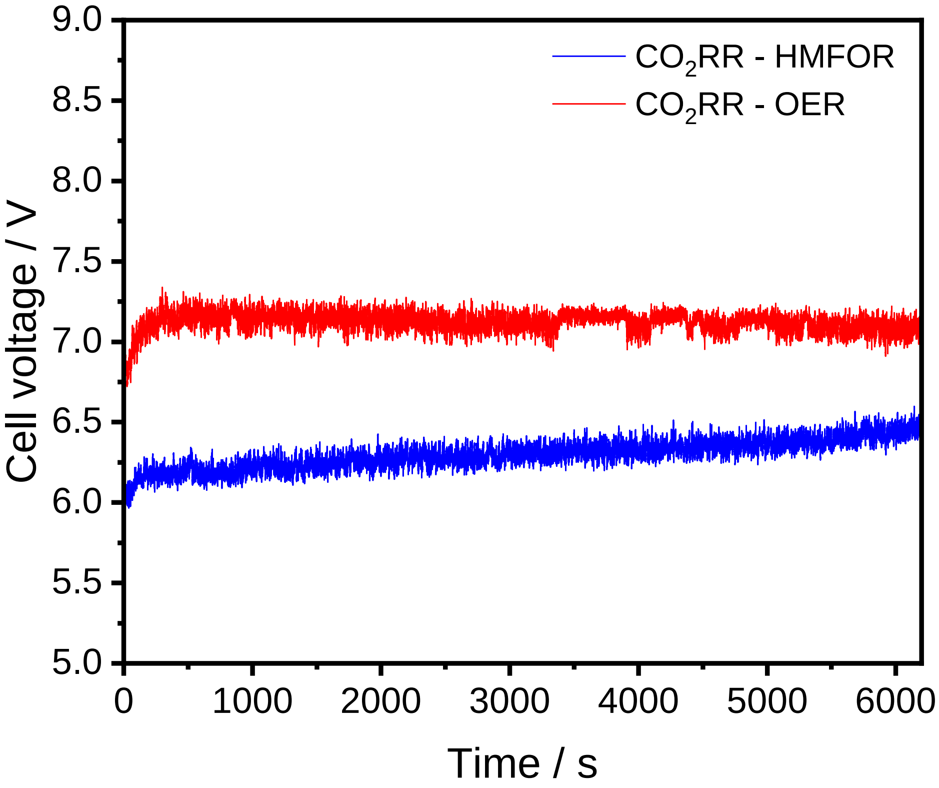

Figure S20: *Two-electrode flow cell setup for a CP experiment at a total current of 0.8 A, conducted with and without HMF Cannizzaro products on the anode side. An Ag/GDE electrode was used as the cathode, while FeCo/NF served as the anode.*

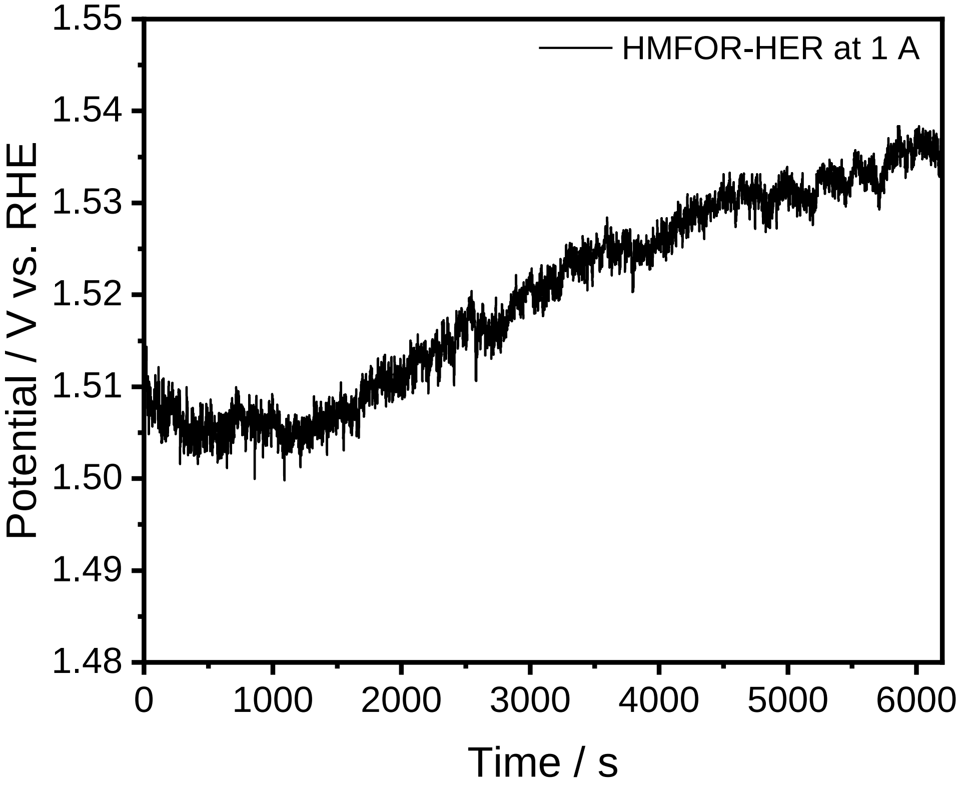

Figure S21: *Three-electrode flow electrolyzer chronopotentiometry (CP) experiment at a total current of 1 A, conducted with HMF Cannizzaro products on the anode side. An Pt foil electrode was used as the cathode, while FeCo/NF served as the anode.*

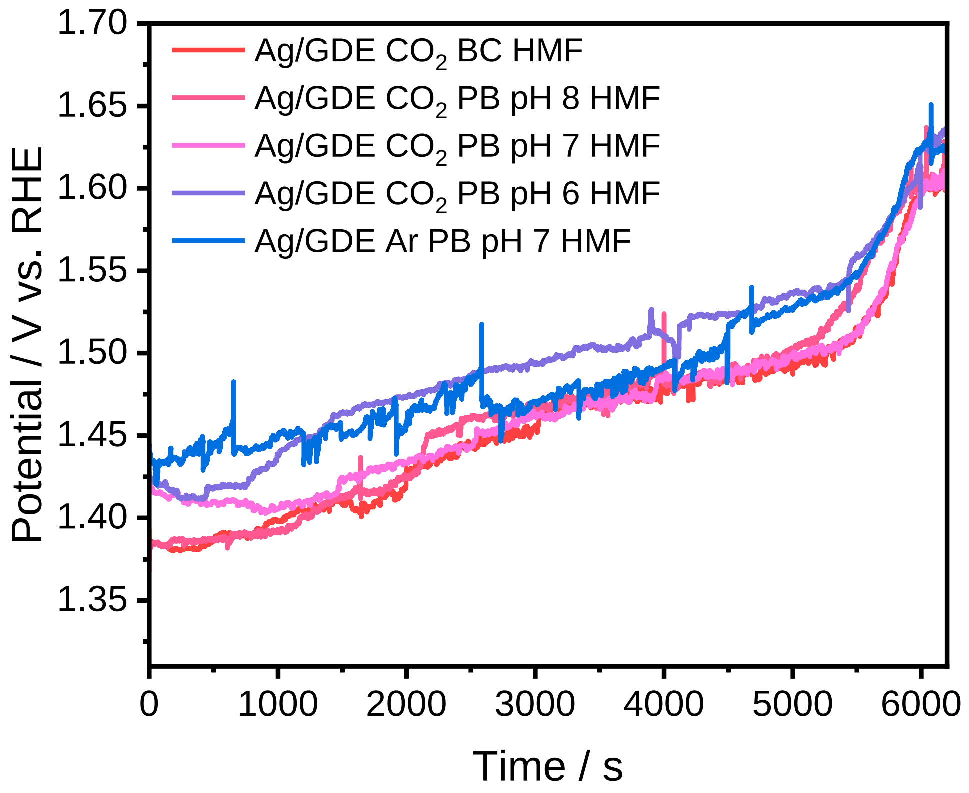

Figure S22: *Chronopotentiometry experiments at 0.8 A for 6200 s. Anode: 5 M KOH in the presence of 500 mM Cannizzaro products (based on initial HMF input before Cannizzaro conversion). Cathode: Bicarbonate buffer (0.5 M $KHCO_3$) or phosphate buffer (2 M), evaluated at varying pH levels.*

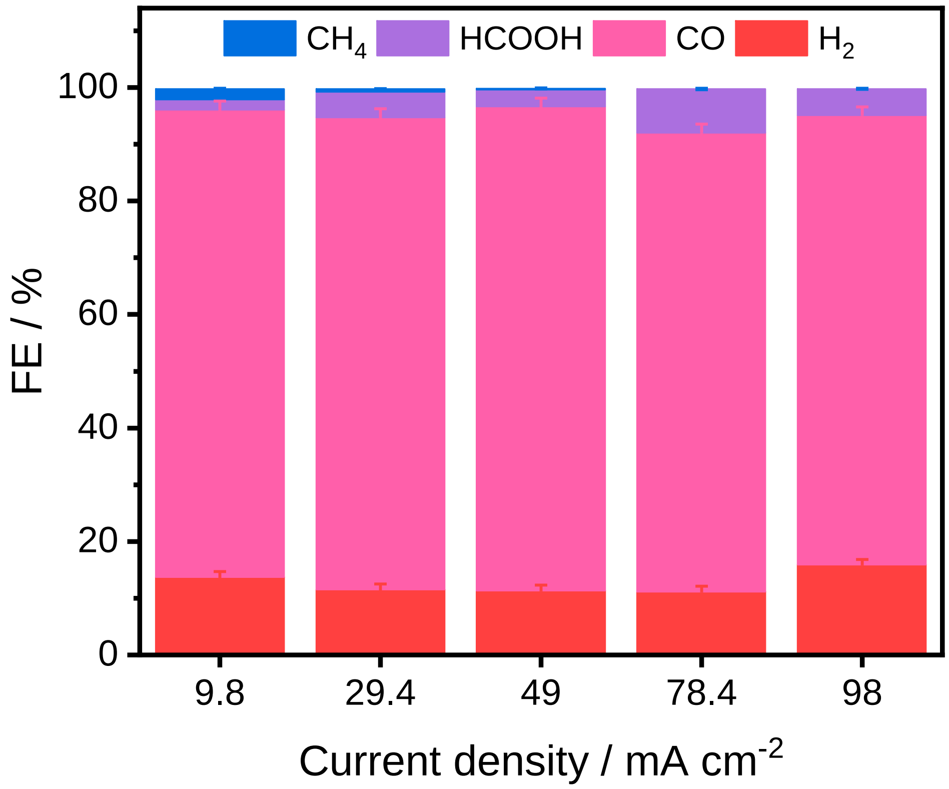

Figure S23: *Three electrode flow cell setup. FE of cathodic products on the silver gas diffusion electrode during the CO_2 reduction reaction coupled with the oxygen evolution reaction (ECO₂RR-OER) at different current densities.*

2.11. Ag/GDE characterization

The morphology and chemical composition of the GDE/catalyst assembly, before and after CO_2RR, were analyzed using scanning electron microscopy (SEM), energy-dispersive X-ray spectroscopy (EDX), and X-ray photoelectron spectroscopy (XPS). Low-magnification SEM images (Figure S26) confirm the uniform distribution of the Ag nanoparticle layer on the GDE substrate. A high-magnification SEM image in Figure S24 provides detailed morphological insights into the deposited catalyst particles, revealing that individual silver nanoparticles, approximately 100 nm in size, agglomerate to form structures resembling 1 µm-sized spheres. EDX analysis, shown in Figure S24, indicates the presence of carbon from the substrate and chlorine from the Sustainion ionomer binder, in addition to silver. SEM images and corresponding elemental maps of the materials after CO_2RR are presented in Figure S25. The catalyst demonstrated good stability during electrolysis, with no discernible morphological changes. Post-electrolysis EDX analysis also detected potassium, attributed to the precipitation of $KHCO_3$ salt on the GDE. XPS analysis (Figure S27) identified silver, chlorine from the Sustainion ionomer, and carbon, fluorine, and oxygen from the GDE substrate. The catalyst stability post-CO_2RR is further supported by the absence of any shift in the silver peak (Figure S27b). The C 1s spectrum (Figure S27c) shows increased contributions from C–O and C=C/C–H components after CO_2RR, while the O 1s spectrum (Figure S27d) exhibits broadening due to an increased H_2O component.

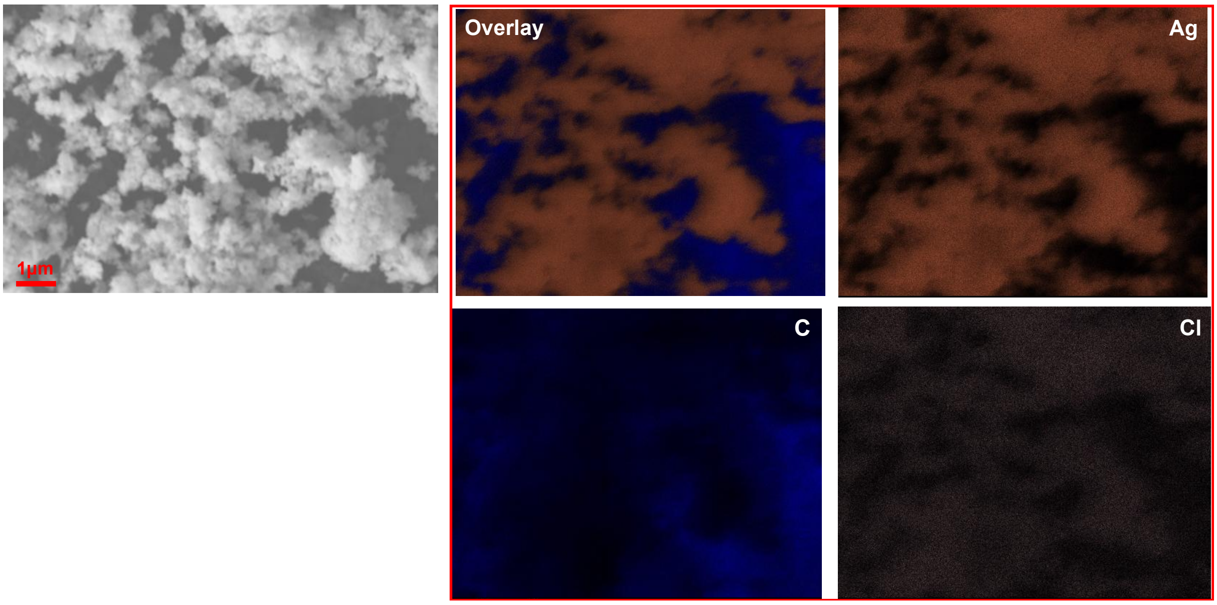

Figure S24: *SEM image along with elemental mapping images of as prepared Ag/GDE.*

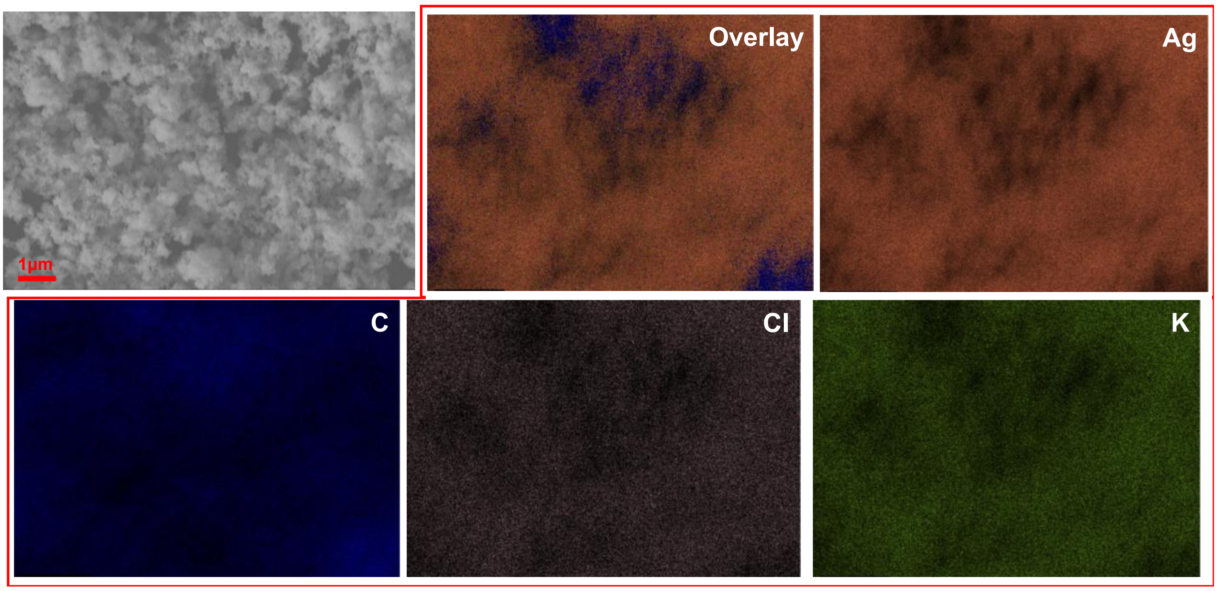

Figure S25: *SEM image along with elemental mapping images of as Ag/GDE post CO_2RR.*

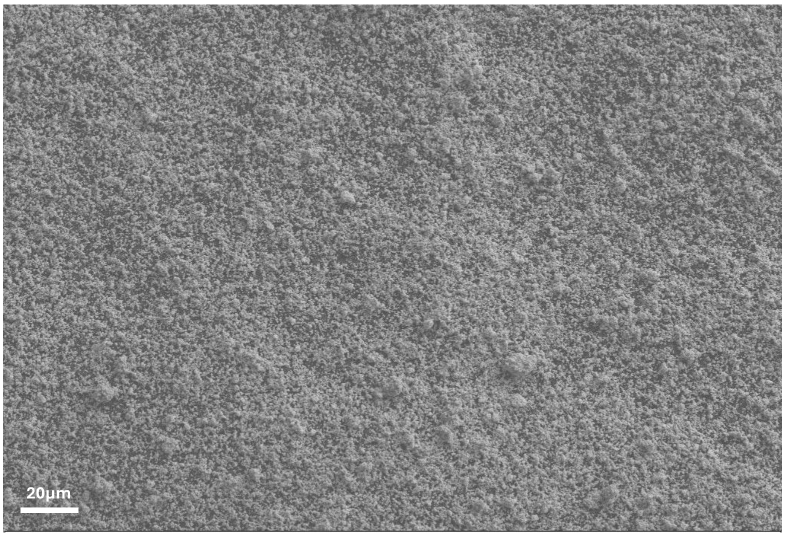

Figure S26: *Low magnification SEM images of Ag-GDE as prepared (pre-CO2RR).*

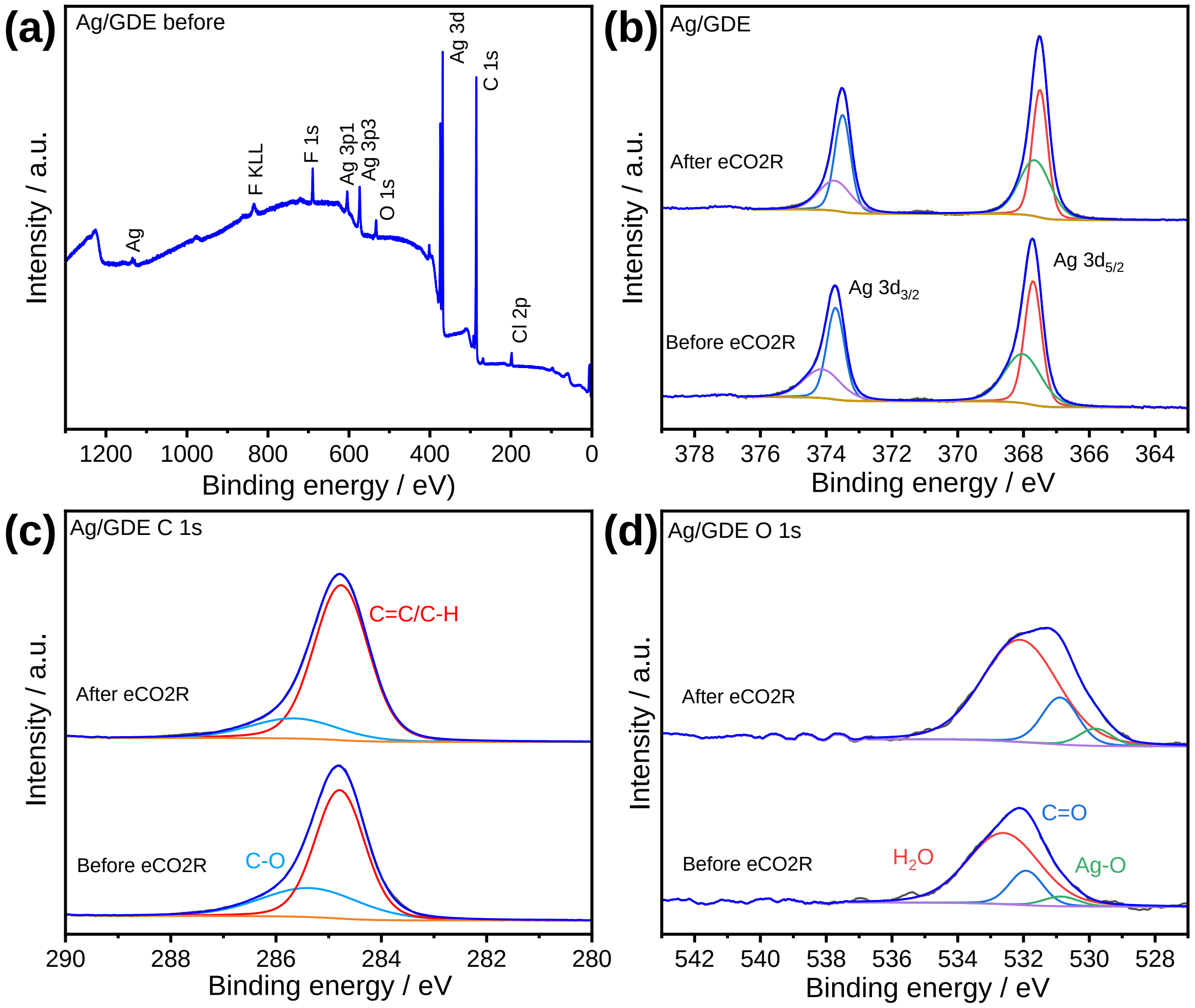

***Figure S27**: XPS spectra for the silver nanoparticles deposited on the gas diffusion electrode (GDE) (a) Ag/GDE as prepared, (b) Ag/GDE 3d, (b) Ag/GDE C 1s and (d) Ag/GDE O 1s before electrochemical reduction of CO2 and post CO_2RR.*

3. Estimation of Syngas and FDCA production

The techno economic analysis (TEA) was performed using a standard discounted cash flow (DCF) approach to evaluate the economic viability of the integrated CO_2RR–HMFOR system. The model accounts for both capital expenditures (CAPEX) and annual operating expenditures (OPEX), including electricity, feedstock, product purification, compression, and maintenance costs. Net cash flows were computed over a 20-year project lifetime, incorporating membrane and electrode replacement at defined intervals. All future cash flows were discounted at a fixed annual rate of 8% to reflect the time value of money. Annual product output was estimated from electrochemical parameters, including current density, electrode area, Faradaic efficiency, and the number of electrons transferred per molecule. Market prices of FDCA and syngas were used to determine revenue, and net present value (NPV) was calculated as the sum of all discounted net cash flows. The model does not include potential carbon credits or tax incentives. All economic inputs and base-case assumptions are summarized in Table S3.

Production Rate:

The production rate of a chemical product from an electrochemical process is related to the applied current, Faradaic efficiency, number of electrons per product molecule, and molar mass. The mass production rate (in kg s^{-1}) is given by:

$$m_{prod} = \frac{I \cdot FE_{prod}}{n_e F} M_{prod} \quad (3.1)$$

Where:

I: Total current [A]

FE_{prod}: Faradaic efficiency for the product [–]

n_e: Number of electrons per mole of product [mol^{-1}]

F: Faraday constant = 96485 C/mol

M_{prod}: Molar mass of the product [kg/mol]

Total Discounted Costs ($): The total discounted cost represents the present value of all cash flows over the project lifetime, accounting for both capital and operational expenses, offset by any revenue. It is calculated as:

$$C_{disc} = \sum_{t=0}^{n} \frac{CAPEX_t + OPEX_t - Revenue_t}{(1+i)^t} \quad (3.2)$$

Where:

- i: Discount rate
- t: Year
- n: Project lifetime [years]
- $CAPEX_t$: Capital expenditure in year t [$]
- $OPEX_t$: Operating expenditure in year t [$]
- $Revenue_t$: Revenue in year t [$]

Net Cash Flow (NCF): The net cash flow is the difference between total revenue and total costs (CAPEX and OPEX) in a given year:

$$CF_t = Revenue_t - (CAPEX_t + OPEX_t) \quad (3.3)$$

Discounted Cash Flow (DCF): Discounted cash flow represents the value of net cash flow adjusted for the time value of money:

$$DCF_t = \frac{CF_t}{(1+i)^t} \quad (3.4)$$

Net Present Value (NPV): Net Present Value (NPV) is the cumulative value of all discounted net cash flows over the entire project lifetime. It indicates the total value generated (or lost) by the project when future cash flows are adjusted to present-day terms:

$$NPV = \sum_{t=0}^{n} \frac{CF_t}{(1+i)^t} \quad (3.5)$$

Where:

- CF_t: Net cash flow in year t [$]
- i : Discount rate
- n: Project lifetime [years]

Annuity Factor (AF): The annuity factor is used to convert a present value (like discounted cost) into an equivalent annual value, accounting for time value of money over the project life:

$$AF = \frac{i}{1-(1+i)^{-n}} \quad (3.6)$$

Where:

- i: Discount rate
- n: Project lifetime [years]

Annual Production (tons/year): The annual production is converted from mass flow rate, and lifetime production is the product of annual output and project duration:

$$P_{annual} = \frac{m_{kg}}{1000} \quad (3.7)$$

Lifetime Production (tons):

$$P_{lifetime} = P_{annual} \cdot n \quad (3.8)$$

Revenue Share (FDCA): The FDCA revenue share is the fraction of total revenue attributed to FDCA:

$$R_{FDCA} = \frac{m_{FDCA} \cdot p_{FDCA}}{\text{Total Revenue}} \tag{3.9}$$

Levelized Cost of Production (LCOP): The LCOP distributes the total discounted cost over the lifetime product output, weighted by revenue share:

$$LCOP = \frac{C_{disc} \cdot R}{P_{lifetime}} \tag{3.10}$$

Alternate (Annuity-Based):

$$LCOP = \frac{C_{disc} \cdot R}{AF \cdot P_{annual}} \tag{3.11}$$

Table S3: *Base case assumptions andiInputs.*

Parameter	Value	Unit	Ref.
Cell Voltage	2.7	V	[23]
Current Density	200	mA/cm^2	[24]
Electrode Area	72.5	m^2	Calculated from target annual FDCA production (~1000 ton/year) using reported methods for CO_2RR TEA[25-26]
Faradaic Efficiency (FDCA)	0.89	-	This study
Faradaic Efficiency (Syngas)	0.92	-	This study
Annual Operating Time	8000	hours/year	[25]
Membrane Cost	800	$\$/m^2$	[27]
Membrane Lifetime	2	years	[28]
Electrode Replacement Cost	1000	$\$/m^2$	[25]
Electrode Lifetime	5	years	[28]
Electricity Price	0.05	$/kWh	[29]
Project Lifetime	20	years	[29]
Discount Rate	8	%	[25]
CAPEX	10000	$\$/m^2$	[23]
FDCA Market Price	1800	$/ton	[30]
Syngas Market Price	100	$/ton	[31]

Table S4: *Calculated outputs.*

Parameter	Value	Unit	Ref.
Total current (A)	145000.00	A	
Annual FDCA (kg)	1002172.19	kg/year	
Annual Syngas (kg)	298641.24	kg/year	
Annual Revenue ($)	1863638.19	$	
Electricity cost ($)	156600.00	$	
HMF cost ($)	962085.30	$	
FDCA purification ($)	50108.61	$	calculated from 0.05$/kg purification cost in ref [30]
Syngas compression ($)	5972.82	$	calculated from 0.02$/kg compression in ref cost [28]
Maintenance cost ($)	21750.00	$	3% of total capex cost per year in ref [25]
Total Annual OPEX ($)	1196516.74	$	

Table S5: Yearly cash flow.

Year	CAPEX ($)	Membrane Rep ($)	Electrode Rep ($)	Annual OPEX ($)	Revenue ($)	Net Cash Flow ($)
**0**	-725000	0	0	0	0	-725000
**1**	0	0	0	-1196516.739	1863638.195	667121.4553
**2**	0	-58000	0	-1254516.739	1863638.195	609121.4553
**3**	0	0	0	-1196516.739	1863638.195	667121.4553
**4**	0	-58000	0	-1254516.739	1863638.195	609121.4553
**5**	0	0	-72500	-1269016.739	1863638.195	594621.4553
**6**	0	-58000	0	-1254516.739	1863638.195	609121.4553
**7**	0	0	0	-1196516.739	1863638.195	667121.4553
**8**	0	-58000	0	-1254516.739	1863638.195	609121.4553
**9**	0	0	0	-1196516.739	1863638.195	667121.4553
**10**	0	-58000	-72500	-1327016.739	1863638.195	536621.4553
**11**	0	0	0	-1196516.739	1863638.195	667121.4553
**12**	0	-58000	0	-1254516.739	1863638.195	609121.4553
**13**	0	0	0	-1196516.739	1863638.195	667121.4553
**14**	0	-58000	0	-1254516.739	1863638.195	609121.4553
**15**	0	0	-72500	-1269016.739	1863638.195	594621.4553
**16**	0	-58000	0	-1254516.739	1863638.195	609121.4553
**17**	0	0	0	-1196516.739	1863638.195	667121.4553
**18**	0	-58000	0	-1254516.739	1863638.195	609121.4553
**19**	0	0	0	-1196516.739	1863638.195	667121.4553
**20**	0	-58000	-72500	-1327016.739	1863638.195	536621.4553
					Total ($)	11747429.11

Table S6: NPV comparison (upon replacing OER with HMFOR).

Flow cell (this study)	Cell voltage (V)	Current density (mA/cm2)	NPV ($)	Change when replaced OER by HMFOR (%)
CO2RR-OER	7.1	80	1439371.64	
CO2RR-HMFOR	6.2	80	1856971.64	
Change in NPV (in million $) upon replacing OER with HMFOR			0.42	29%
MEA[23]				
CO2RR-OER	3	200	11399429.11	
CO2RR-HMFOR	2.7	200	11747429.11	
Change in NPV (in million $) upon replacing OER with HMFOR			0.35	3%

Table S7: HMF cost sensitivity analysis.

HMF price (P_HMF/ton)	HMF input annual cost ($)	Change HMF input annual cost (%)	Total annual OPEX ($)	Change Total annual OPEX (%)	NPV ($)	Change in NPV (%)
500	601303.32	-37.50	835734.75	-30.15	18963068.89	61.42
800	962085.30	0.00	1196516.74	0.00	11747429.11	0.00
1200	1443127.96	50.00	1677559.39	40.20	2126576.06	-81.90
1289	1550159.95	61.13	1784591.38	49.15	-14063.75	-100.12
2000	2405213.26	150.00	2639644.70	120.61	-17115130.04	-245.69
3000	3607819.89	275.00	3842251.33	221.12	-41167262.66	-450.44

Table S8: HMF cost sensitivity analysis.

Carbon balance (%)	HMF input annual cost ($)	Change HMF input annual cost (%)	Total annual OPEX ($)	Change total annual OPEX (%)	NPV ($)	Change in NPV (%)
100.00	962085.30	0.00	1196516.74	0.00	11747429.11	0.00
90.00	1058293.84	10.00	1292725.27	8.04	9823258.50	-16.38
80.00	1154502.37	20.00	1388933.80	16.08	7899087.89	-32.76
70.00	1250710.90	30.00	1485142.33	**24.12**	5974917.28	-49.14
60.00	1346919.43	40.00	1581350.86	**32.16**	4050746.67	-65.52
50.00	1443127.96	50.00	1677559.39	40.20	2126576.06	-81.90
40.00	1539336.49	60.00	1773767.92	48.24	202405.45	-98.28
30.00	1635545.02	70.00	1869976.45	56.29	-1721765.16	-114.66
20.00	1731753.55	80.00	1966184.98	64.33	-3645935.77	-131.04

4. References

[1] C. Wang, Y. Wu, A. Bodach, M. L. Krebs, W. Schuhmann, F. Schüth, A Novel Electrode for Value-Generating Anode Reactions in Water Electrolyzers at Industrial Current Densities, *Angew. Chem. Int. Ed.* **2023**, *62*, e202215804.

[2] E. Budiyanto, S. Salamon, Y. Wang, H. Wende, H. Tüysüz, Phase Segregation in Cobalt Iron Oxide Nanowires toward Enhanced Oxygen Evolution Reaction Activity, *JACS Au* **2022**, *2*, 697-710.

[3] K. Kawashima, R. A. Márquez, Y. J. Son, C. Guo, R. R. Vaidyula, L. A. Smith, C. E. Chukwuneke, C. B. Mullins, Accurate Potentials of Hg/HgO Electrodes: Practical Parameters for Reporting Alkaline Water Electrolysis Overpotentials, *ACS Catal.* **2023**, *13*, 1893-1898.

[4] D. M. Morales, M. Risch, Seven steps to reliable cyclic voltammetry measurements for the determination of double layer capacitance, *J. Phys. Energy* **2021**, *3*.

[5] N. R. Babij, E. O. McCusker, G. T. Whiteker, B. Canturk, N. Choy, L. C. Creemer, C. V. De Amicis, N. M. Hewlett, P. L. Johnson, J. A. Knobelsdorf, F. Z. Li, B. A. Lorsbach, B. M. Nugent, S. J. Ryan, M. R. Smith, Q. Yang, NMR Chemical Shifts of Trace Impurities: Industrially Preferred Solvents Used in Process and Green Chemistry, *Org. Process Res. Dev.* **2016**, *20*, 661-667.

[6] T. L. Hwang, A. J. Shaka, Water Suppression That Works - Excitation Sculpting Using Arbitrary Wave-Forms and Pulsed-Field Gradients, *J. Magn. Reson., Ser A* **1995**, *112*, 275-279.

[7] M. L. Krebs, A. Bodach, C. L. Wang, F. Schüth, Stabilization of alkaline 5-HMF electrolytes Cannizzaro reaction for the electrochemical oxidation to FDCA, *Green Chem.* **2023**, *25*, 1797-1802.

[8] S. Q. Niu, S. W. Li, Y. C. Du, X. J. Han, P. Xu, How to Reliably Report the Overpotential of an Electrocatalyst, *ACS Energy Lett.* **2020**, *5*, 1083-1087.

[9] Y. Sun, J. Wang, Y. Qi, W. Li, C. Wang, Efficient Electrooxidation of 5-Hydroxymethylfurfural Using Co-Doped Ni_3S_2 Catalyst: Promising for H_2 Production under Industrial-Level Current Density, *Adv. Sci.* **2022**, *9*, e2200957.

[10] S. Q. Li, S. B. Wang, Y. H. Wang, J. H. He, K. Li, Y. J. Xu, M. X. Wang, S. Y. Zhao, X. N. Li, X. Zhong, J. G. Wang, Doped Mn Enhanced NiS Electrooxidation Performance of HMF into FDCA at Industrial-Level Current Density, *Adv. Funct. Mater.* **2023**, *33*.

[11] D. Chen, Y. Ding, X. Cao, L. Wang, H. Lee, G. Lin, W. Li, G. Ding, L. Sun, Highly Efficient Biomass Upgrading by a Ni-Cu Electrocatalyst Featuring Passivation of Water Oxidation Activity, *Angew. Chem. Int. Ed.* **2023**, *62*, e202309478.

[12] R. Zhang, S. Jiang, Y. Rao, S. Chen, Q. Yue, Y. Kang, Electrochemical biomass upgrading on CoOOH nanosheets in a hybrid water electrolyzer, *Green Chem.* **2021**, *23*, 2525-2530.

[13] N. Jiang, B. You, R. Boonstra, I. M. T. Rodriguez, Y. J. Sun, Integrating Electrocatalytic 5-Hydroxymethylfurfural Oxidation and Hydrogen Production via Co-P-Derived Electrocatalysts, *ACS Energy Lett.* **2016**, *1*, 386-390.

[14] Y. Lu, T. Liu, C. L. Dong, Y. C. Huang, Y. Li, J. Chen, Y. Zou, S. Wang, Tuning the Selective Adsorption Site of Biomass on Co_3O_4 by Ir Single Atoms for Electrosynthesis, *Adv. Mater.* **2021**, *33*, e2007056.

[15] J. Wang, Z. Zhao, C. Shen, H. Liu, X. Pang, M. Gao, J. Mu, F. Cao, G. Li, Ni/NiO heterostructures encapsulated in oxygen-doped graphene as multifunctional electrocatalysts for the HER, UOR and HMF oxidation reaction, *Catal. Sci. Technol.* **2021**, *11*, 2480-2490.

[16] B. Zhou, Y. Li, Y. Zou, W. Chen, W. Zhou, M. Song, Y. Wu, Y. Lu, J. Liu, Y. Wang, S. Wang, Platinum Modulates Redox Properties and 5-Hydroxymethylfurfural Adsorption Kinetics of $Ni(OH)_2$ for Biomass Upgrading, *Angew. Chem. Int. Ed.* **2021**, *60*, 22908-22914.

[17] B. Zhou, C.-L. Dong, Y.-C. Huang, N. Zhang, Y. Wu, Y. Lu, X. Yue, Z. Xiao, Y. Zou, S. Wang, Activity origin and alkalinity effect of electrocatalytic biomass oxidation on nickel nitride, *J. Energy Chem.* **2021**, *61*, 179-185.
[18] S. Barwe, J. Weidner, S. Cychy, D. M. Morales, S. Dieckhofer, D. Hiltrop, J. Masa, M. Muhler, W. Schuhmann, Electrocatalytic Oxidation of 5-(Hydroxymethyl)furfural Using High-Surface-Area Nickel Boride, *Angew. Chem. Int. Ed.* **2018**, *57*, 11460-11464.
[19] L. Gao, X. Wen, S. Liu, D. Qu, Y. Ma, J. Feng, Z. Zhong, H. Guan, L. Niu, Nickel-vanadium-cobalt ternary layered double hydroxide for efficient electrocatalytic upgrading of 5-hydroxymethylfurfural to 2,5-furancarboxylic acid at low potential, *J. Mater. Chem. A* **2022**, *10*, 21135-21141.
[20] W. J. Liu, L. N. Dang, Z. R. Xu, H. Q. Yu, S. Jin, G. W. Huber, Electrochemical Oxidation of 5-Hydroxymethylfurfural with NiFe Layered Double Hydroxide (LDH) Nanosheet Catalysts, *ACS Catal.* **2018**, *8*, 5533-5541.
[21] Y. Lu, C. L. Dong, Y. C. Huang, Y. Zou, Z. Liu, Y. Liu, Y. Li, N. He, J. Shi, S. Wang, Identifying the Geometric Site Dependence of Spinel Oxides for the Electrooxidation of 5-Hydroxymethylfurfural, *Angew. Chem. Int. Ed.* **2020**, *59*, 19215-19221.
[22] W. Chen, C. Xie, Y. Wang, Y. Zou, C.-L. Dong, Y.-C. Huang, Z. Xiao, Z. Wei, S. Du, C. Chen, B. Zhou, J. Ma, S. Wang, Activity Origins and Design Principles of Nickel-Based Catalysts for Nucleophile Electrooxidation, *Chem* **2020**, *6*, 2974-2993.
[23] S. Q. Liu, M. R. Gao, S. W. Wu, R. F. Feng, Y. C. Wang, L. F. Cui, Y. Guo, X. Z. Fu, J. L. Luo, A coupled electrocatalytic system with reduced energy input for CO_2 reduction and biomass valorization, *Energy Environ. Sci.* **2023**, *16*, 5305-5314.
[24] Y. Y. Birdja, E. Pérez-Gallent, M. C. Figueiredo, A. J. Göttle, F. Calle-Vallejo, M. T. M. Koper, Advances and challenges in understanding the electrocatalytic conversion of carbon dioxide to fuels, *Nat. Energy* **2019**, *4*, 732-745.
[25] M. Jouny, W. Luc, F. Jiao, General Techno-Economic Analysis of CO_2 Electrolysis Systems, *Ind. Eng. Chem.* **2018**, *57*, 2165-2177.
[26] J. M. Spurgeon, B. Kumar, A comparative technoeconomic analysis of pathways for commercial electrochemical CO_2 reduction to liquid products, *Energy Environ. Sci.* **2018**, *11*, 1536-1551.
[27] M. Heßelmann, H. Minten, T. Geissler, R. G. Keller, A. Bardow, M. Wessling, Why Membranes Matter: Ion Exchange Membranes in Holistic Process Optimization of Electrochemical CO2 Reduction, *Advanced Sustainable Systems* **2023**, *7*.
[28] T. Moore, D. I. Oyarzun, W. Li, T. Y. Lin, M. Goldman, A. A. Wong, S. A. Jaffer, A. Sarkar, S. E. Baker, E. B. Duoss, C. Hahn, Electrolyzer energy dominates separation costs in state-of-the-art CO2 electrolyzers: Implications for single-pass CO2 utilization, *Joule* **2023**, *7*, 782-796.
[29] I. Bagemihl, L. Cammann, M. Perez-Fortes, V. van Steijn, J. R. van Ommen, Techno-economic Assessment of CO(2) Electrolysis: How Interdependencies between Model Variables Propagate Across Different Modeling Scales, *ACS Sustain Chem Eng* **2023**, *11*, 10130-10141.
[30] M. G. Davidson, S. Elgie, S. Parsons, T. J. Young, Production of HMF, FDCA and their derived products: a review of life cycle assessment (LCA) and techno-economic analysis (TEA) studies, *Green Chem.* **2021**, *23*, 3154-3171.
[31] M. Bachmann, S. Völker, J. Kleinekorte, A. Bardow, Syngas from What? Comparative Life-Cycle Assessment for Syngas Production from Biomass, CO_2, and Steel Mill Off-Gases, *ACS Sustain. Chem. Eng.* **2023**, *11*, 5356-5366.
